# Mechanistic insights into SOCS5-related DNA damage and cellular senescence in diabetic retinopathy

**DOI:** 10.1038/s41420-026-03011-3

**Published:** 2026-04-01

**Authors:** Di Yang, Siduo Lu, Hongmei Liu, You Zhou, Hua Zhong

**Affiliations:** Department of Ophthalmology, First Affiliated Hospital of Kunming Medical University, Kunming, China

**Keywords:** Molecular biology, Cell signalling

## Abstract

This study elucidated the mechanistic role of the Suppressor of Cytokine Signaling 5 (SOCS5) in diabetic retinopathy (DR), focusing on DNA damage and cellular senescence pathways. Utilizing both in vitro (high glucose (HG)-induced human retinal microvascular endothelial cells (HRMECs)) and in vivo (streptozotocin-induced DR mouse models) approaches, we demonstrated that SOCS5 was significantly upregulated in DR. SOCS5 knockdown mitigated retinal tissue damage, vascular leakage, and apoptosis in DR mice while reducing DNA damage and cellular senescence in HG-stimulated HRMECs. Mechanistically, SOCS5 promoted DR progression by regulating the expression of Cyclin-Dependent Kinase Inhibitor 1 A (CDKN1A), a key mediator of cell cycle arrest and senescence. Furthermore, we identified POU Class 2 Homeobox 1 (POU2F1) as an upstream transcriptional activator of SOCS5, forming a novel POU2F1-SOCS5-CDKN1A axis that drove DR pathogenesis. Inhibition of POU2F1 and SOCS5 ameliorated DR-related pathology in mice, suggesting a novel therapeutic strategy. These findings reveal a previously unrecognized signaling pathway in DR and highlight SOCS5 as a promising target for intervention.

## Introduction

Diabetic retinopathy (DR) is one of the most common and serious microvascular complications of diabetes mellitus and a leading cause of vision loss among working-age adults [1, 2]. Its pathogenesis is multifactorial, involving mechanisms such as hyperglycaemia-induced oxidative stress, inflammatory responses, vascular permeability dysfunction, and neurodegeneration [3]. Current clinical treatments, including laser photocoagulation and anti-VEGF therapy, can mitigate disease progression but do not restore pre-existing retinal damage. Therefore, a deeper understanding of the key molecular regulators of DR is essential for developing novel and more effective interventional strategies [4, 5].

In recent years, advances in molecular biology, cell biology, and genetics have significantly advanced our understanding of DR pathogenesis [6–8]. DR is recognized as a multifactorial and multistage disease, involving metabolic disorders, inflammation, oxidative stress, apoptosis, and vascular endothelial dysfunction [9, 10]. Despite progress, the precise molecular mechanisms underlying DR remain incompletely defined. Through high-throughput sequencing and bioinformatics analysis, our group identified SOCS5 as a key gene in the disease process of DR and further inferred that both the upstream target of SOCS5, POU2F1, and its downstream molecule, CDKN1A, play important roles [11, 12].

SOCS5 is a crucial negative regulator of cytokine signal transduction and contributes to the maintenance of immune and metabolic homeostasis [13]. Studies have shown that SOCS5 regulates cell proliferation and migration by inhibiting the PI3K/Akt/mTOR pathway and attenuates glomerular injury by regulating oxidative stress and inflammatory responses in diabetic nephropathy [14]. In hepatocellular carcinoma, SOCS5 knockdown inhibits HIF-1α-dependent mitochondrial damage, thereby reducing tumor metastasis [15]. In addition, SOCS5 has been shown to regulate the EGFR signaling pathway in the tumor microenvironment, suggesting that it may influence angiogenesis-related factor expression. However, the role of SOCS5 in DR remains unclear, and whether it participates in DR progression by regulating DNA damage response or senescence processes in retinal cells has yet to be explored.

POU2F1 encodes a DNA-binding transcription factor belonging to an evolutionarily conserved family essential for cell type specification, and its expression dynamics during development are critical for proper embryonic differentiation [16]. POU2F1 is regulated by a variety of genes and participates in processes such as embryonic development, immune and inflammatory responses, and energy metabolism [17]. However, the specific role of POU2F1 in DR remains unexplored.

DNA damage and cellular senescence are closely linked and serve as key pathogenic factors in DR, in which SOCS5 plays a critical role [18]. DNA damage can activate intracellular stress responses, leading to cell cycle arrest, apoptosis, or senescence. In DR patients, chronic hyperglycemia and metabolic dysfunction enhance retinal cell susceptibility to DNA damage [19]. Clinical studies indicate that DNA damage levels in peripheral blood mononuclear cells correlate positively with the severity of DR. Moreover, sustained DNA damage activates the p53/p21 and p16 pathways, induces endothelial cell senescence, and releases proinflammatory factors through senescence-associated secretory phenotypes (SASPs), which further disrupts the blood-retinal barrier and promotes pathologic neovascularization at the same time [20]. Therefore, understanding how SOCS5 modulates DNA damage and senescence is essential for clarifying DR pathogenesis.

This study aims to elucidate the mechanism by which SOCS5 contributes to DR progression through DNA damage and cellular senescence pathways. By establishing in vitro and in vivo models and employing multidisciplinary approaches, including molecular biology, cell biology, and animal experimentation, this work systematically investigated SOCS5 role in DR pathogenesis and its underlying molecular mechanisms. Our results suggest that targeted inhibition of SOCS5 or POU2F1 significantly improves DR-related pathology, providing a potential therapeutic strategy [21]. Furthermore, this study lays a foundation for future research on SOCS5 as a biomarker for early diagnosis and a druggable target for DR treatment.

## Results

### Identification of SOCS5 as a key gene in human DR progression

**Fig. 1:**
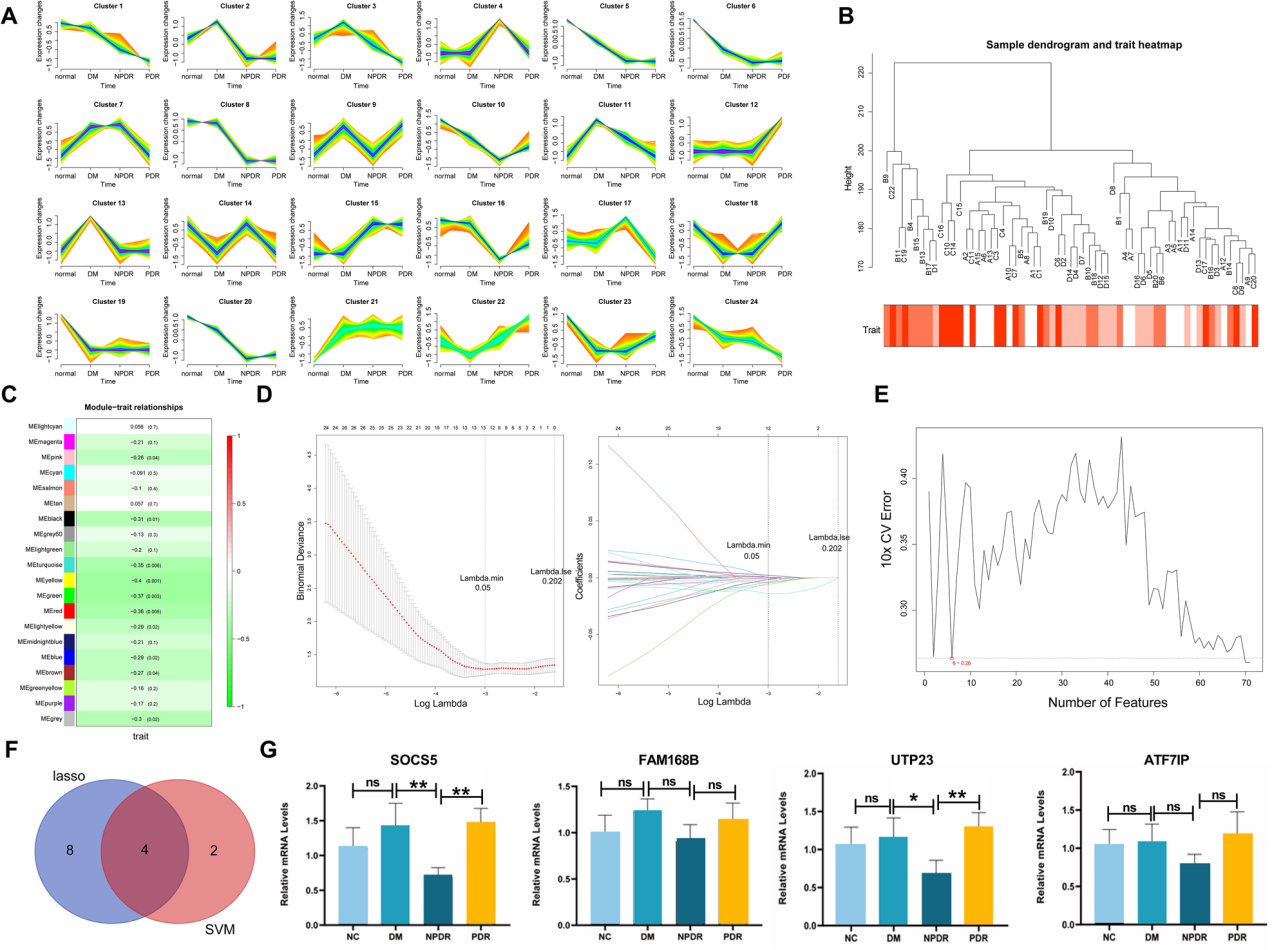
SOCS5 was identified as a key gene during human DR progression. **A** Trend analysis of transcriptomic data from human blood samples (NC, DM, NPDR, PDR). **B** Sample dendrogram and trait heatmap from WGCNA. The clinical traits are color‑coded by intensity, with lighter red indicating lower values and darker red indicating higher values. **C** Module-trait relationships from WGCNA. **D** LASSO regression analysis for feature selection. **E** SVM-RFE algorithm analysis. **F** Venn diagram showing the intersection of key genes identified by LASSO regression and SVM-RFE. **G** qRT-PCR validation of SOCS5, FAM168B, UTP23, and ATF7IP mRNA levels in peripheral blood samples from NC, DM, NPDR, and PDR subjects. A one-way ANOVA test was used for multiple variable comparison. *p ˂ 0.05, **p ˂ 0.01, ***p ˂ 0.001 vs DM/ NPDR. ns means no significant difference vs NC/DM/NPDR.

Bioinformatics analysis was conducted using transcriptomic data from human whole blood samples. Trend analysis identified gene clusters (cluster1, cluster5, cluster6, and cluster24) whose expression patterns followed the Normal→DM→NPDR→PDR progression (Fig. 1A). Subsequent WGCNA and machine learning approaches (LASSO regression and SVM-RFE) were employed to pinpoint hub genes (Fig. 1B–E). The intersection of genes identified by LASSO and SVM-RFE yielded four key candidates: SOCS5, FAM168B, UTP23, and ATF7IP (Fig. 1F). To validate these findings, peripheral blood samples from an independent cohort were analyzed. qRT-PCR analysis demonstrated a significant increase in SOCS5 mRNA expression in the PDR group relative to the NPDR group (Fig. 1G). These results identified SOCS5 as a key gene during human DR progression.

### SOCS5 was upregulated in both in vivo and in vitro models of DR

**Fig. 2:**
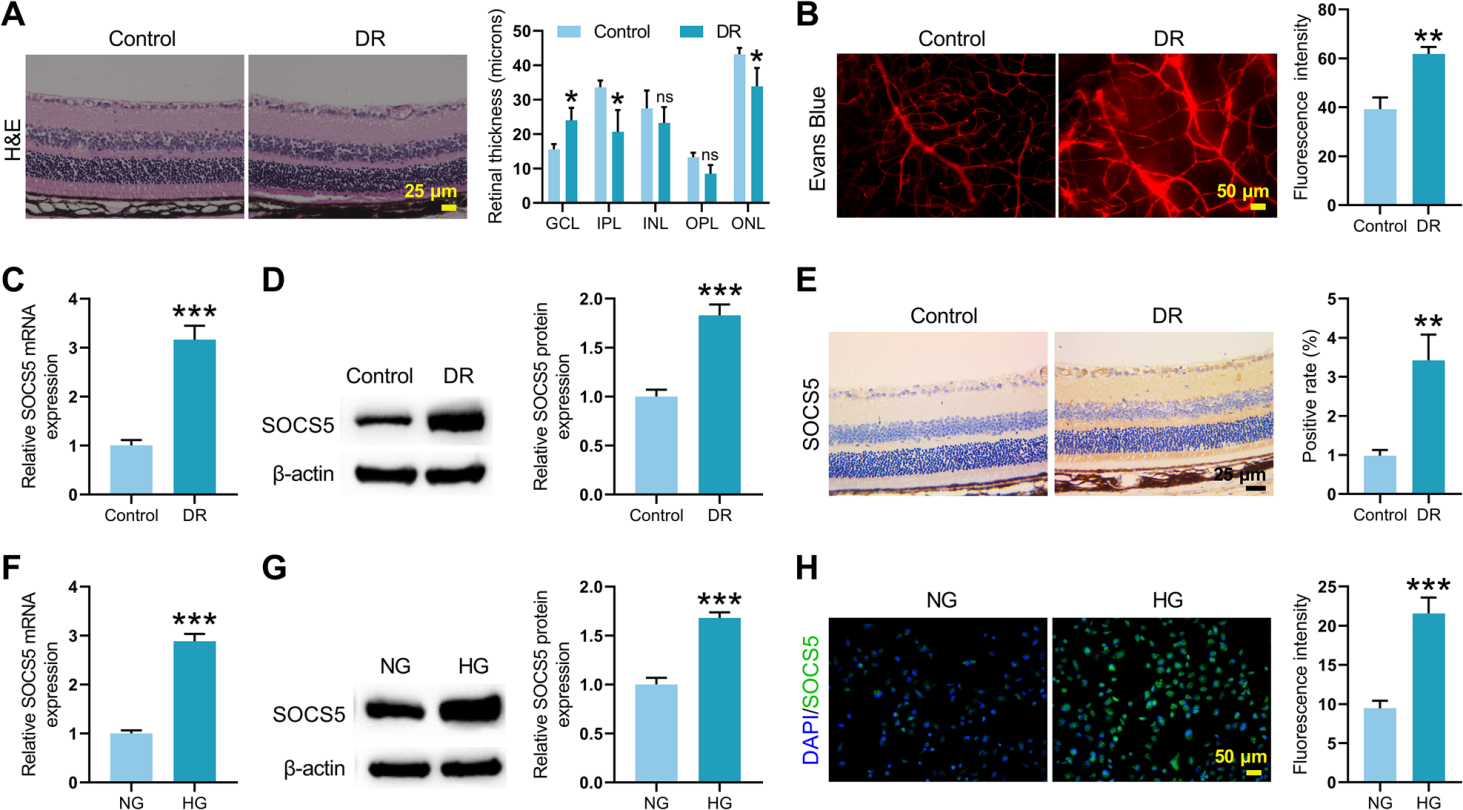
SOCS5 expression was assessed in the retinas of DR mice and in HG-induced HRMECs. Mice were randomly divided into a Control group and a DR group, with 9 mice in each group. A DR mouse model was established by feeding a high-fat diet and inducing with STZ. **A** H&E staining analysis of histopathological changes in the retina of mice from control and DR mice. **B** Evans Blue staining of retinal sections assessing morphological changes in retinal vascular damage. **C**, **D** qRT-PCR and WB were performed to detect SOCS5 mRNA and protein level in retinal tissues. **E** Immunohistochemical staining of SOCS5 expression in retinal tissues. HRMECs were cultured under NG or HG conditions for 48 h. **F**,**G** qRT-PCR and WB evaluating SOCS5 mRNA and protein level in HRMECs cells from different treatment groups. **H** Immunofluorescence staining of SOCS5 expression (green) in HRMECs cells. Nuclei were counterstained with DAPI (blue). Student’s *t* tests were used for comparing two variables. Data are from n independent biological replicates (**A**–**E**, *n* = 3 mice; **F-H**, *n* = 3 independent cultures). *p ˂ 0.05, **p ˂ 0.01, ***p ˂ 0.001 vs Control/ NG. ns means no significant difference vs Control.

To investigate SOCS5 functional role in DR pathogenesis, we established a DR mouse model using C57BL/6 mice fed a high-fat/high-sucrose diet followed by STZ administration. After enucleation, retinal tissues were isolated for analysis. Histopathological examination revealed significant thickening of the ganglion cell layer and aberrant retinal vascular morphology, confirming retinal tissue damage (Fig. 2A, B). SOCS5 expression was significantly upregulated in the DR group at both mRNA and protein levels (Fig. 2C, D). Immunohistochemistry further confirmed an increase in SOCS5-positive cells in the retinal tissue of the DR group (Fig. 2E).

Consistently, HG‑treated HRMECs exhibited elevated SOCS5 expression, as demonstrated by qRT-PCR and WB (Fig. 2F, G). Immunofluorescence staining also showed enhanced SOCS5 fluorescence signal in HG-stimulated HRMECs (Fig. 2H). These results indicate that SOCS5 is upregulated in DR and may contribute to its pathogenesis.

### SOCS5 knockdown alleviated DNA damage and cellular senescence in DR

**Fig. 3:**
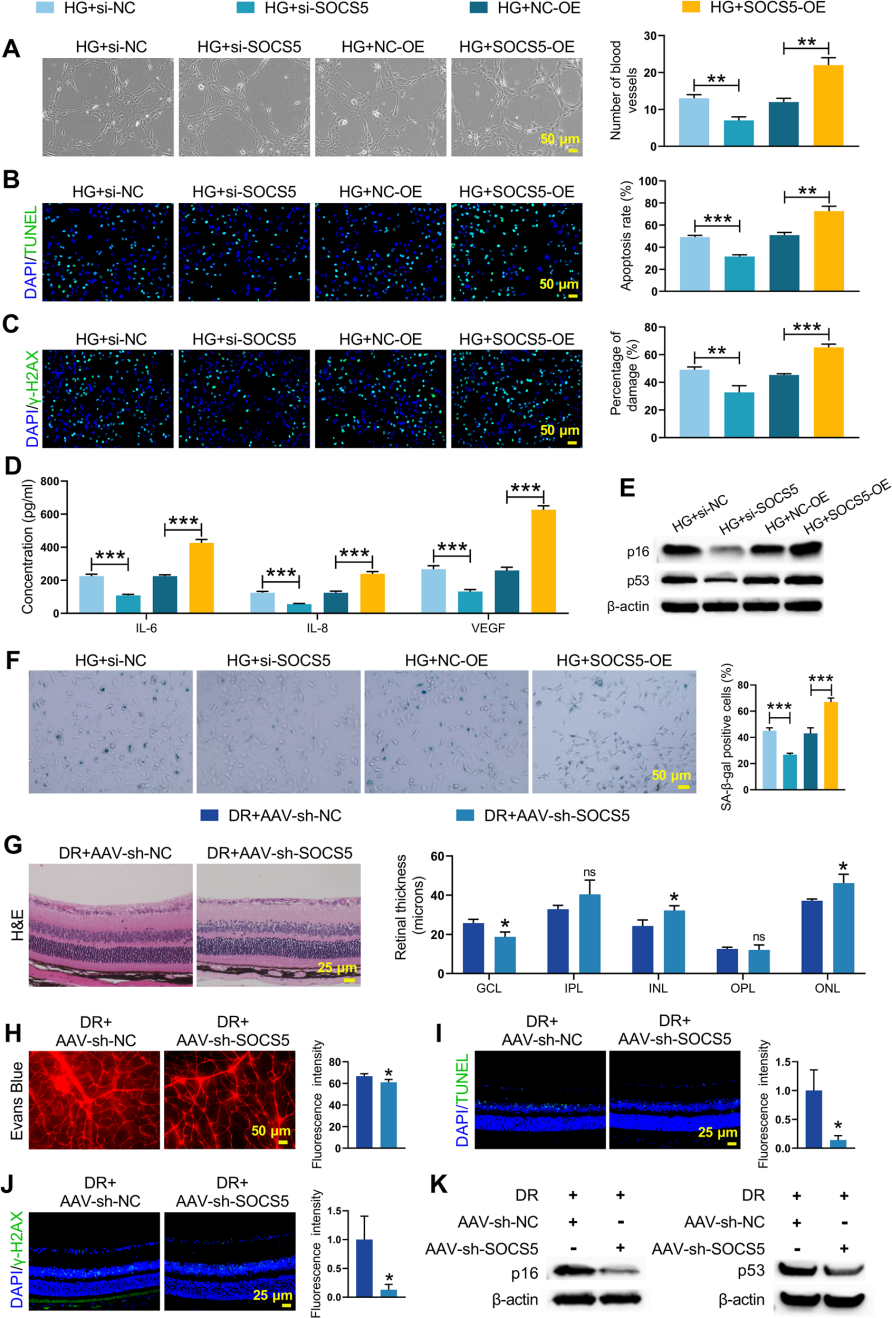
SOCS5 knockdown attenuated, while its overexpression exacerbated HG-induced DNA damage and cellular senescence in HRMECs and retinal damage in DR mice. HG-induced HRMECs were transfected with si-SOCS5, si-NC, SOCS5-OE, or NC-OE for 24 h followed by HG stimulation for 48 h. **A** Matrigel tubule formation assay was performed to assess intracellular vessel formation in HRMECs. **B** TUNEL was used to detect apoptosis in HRMECs cells. **C** γ-H2AX immunofluorescence was conducted to evaluate intracellular DNA damage in HRMECs. **D** ELISA analysis of intracellular senescence-associated secretory phenotypes IL-6, IL-8, and VEGF in HRMECs. **E** WB was utilized to measure the expression of cellular senescence-related molecules p53 and p16 in HRMECs. **F** Senescence-associated SA-β-gal staining assessing intracellular senescence in HRMECs. STZ-induced DR mice were intravitreally injected with AAV-sh-NC or AAV-sh-SOCS5 (*n* = 9 mice per group). **G** H&E staining was used to analyze the effect of sh-SOCS5 on histopathological changes in the retina of DR mice. **H** Evans Blue staining of retinal vasculature demonstrating the effect of sh-SOCS5 on retinal vascular damage of DR mice. **I** TUNEL detection showing the impact of sh-SOCS5 on apoptosis in DR mouse retinal tissue. **J** γ-H2AX immunofluorescence assay assessing the effect of sh-SOCS5 on DNA damage in DR mouse retinal tissue. **K** WB analysis of p53 and p16 expression in retinal tissues, exhibiting the influence of sh-SOCS5 on the molecules related to retinal tissue senescence. Student’s *t* tests were used for comparing two variables. A one-way ANOVA test was used for multiple variable comparison. Data are from n independent biological replicates (**A**–**F**, *n* = 3 independent cultures; **G**–**K**, *n* = 3 mice). *p ˂ 0.05, **p ˂ 0.01, ***p ˂ 0.001 vs HG+si-NC/ DR + AAV-sh-NC. ns means no significant difference vs DR + AAV-sh-NC group.

To explore the functional effects of SOCS5 in DR, HRMECs were treated with SOCS5 overexpression or knockdown, followed by HG stimulation. The transfection efficiency of SOCS5 knockdown was validated (Supplementary Fig. 1A, B). In HG-stimulated HRMECs, SOCS5 overexpression increased its mRNA and protein levels, whereas knockdown decreased them (Supplementary Fig. 2A, B). SOCS5 knockdown significantly impaired tubule formation capacity, while SOCS5 overexpression enhanced angiogenesis (Fig. 3A). TUNEL assay and immunofluorescence staining for γ-H2AX and 8-oxo-dG confirmed that SOCS5 knockdown decreased cellular apoptosis and DNA damage in HG-stimulated HRMECs, whereas SOCS5 overexpression exacerbated these effects (Fig. 3B, C and Supplementary Fig. 2C). ELISA analysis revealed that SOCS5 knockdown reduced secretion of senescence-associated factors (IL-6, IL-8, and VEGF) in HG-stimulated HRMECs, while its overexpression increased their production (Fig. 3D). Correspondingly, WB analysis demonstrated that senescence markers p53 and p16 levels in HG-stimulated HRMECs were reduced following SOCS5 knockdown and elevated after its overexpression (Fig. 3E and Supplementary Fig. 2D). SA-β-gal staining confirmed that SOCS5 knockdown reduced cellular senescence in HG-stimulated HRMECs, while its overexpression increased senescence (Fig. 3F).

To further confirm the role of SOCS5 in vivo, SOCS5 was knocked down in DR mice using adeno-associated virus. Knockdown efficiency was confirmed (Supplementary Fig. 1C, D). qRT-PCR, WB and immunohistochemical detection showed that sh-SOCS5 treatment reduced SOCS5 expression in retinal tissues of DR mice (Supplementary Fig. 2E–G). Histopathological analysis confirmed that sh-SOCS5 treatment ameliorated pathological features in retinal tissues of DR mice, including ganglion cell layer thickening and vascular abnormalities (Fig. 3G, H). Moreover, TUNEL and γ-H2AX staining indicated that sh-SOCS5 reduced apoptosis and DNA damage in retinal tissues of DR mice (Fig. 3I, J). WB analysis showed decreased p53 and p16 levels in retinal tissues of DR mice after treatment with sh-SOCS5 (Fig. 3K and Supplementary Fig. 2H). These results demonstrate that SOCS5 regulates retinal damage through mechanisms involving DNA damage and senescence.

### SOCS5 upregulated CDKN1A to promote DNA damage and cellular senescence

**Fig. 4:**
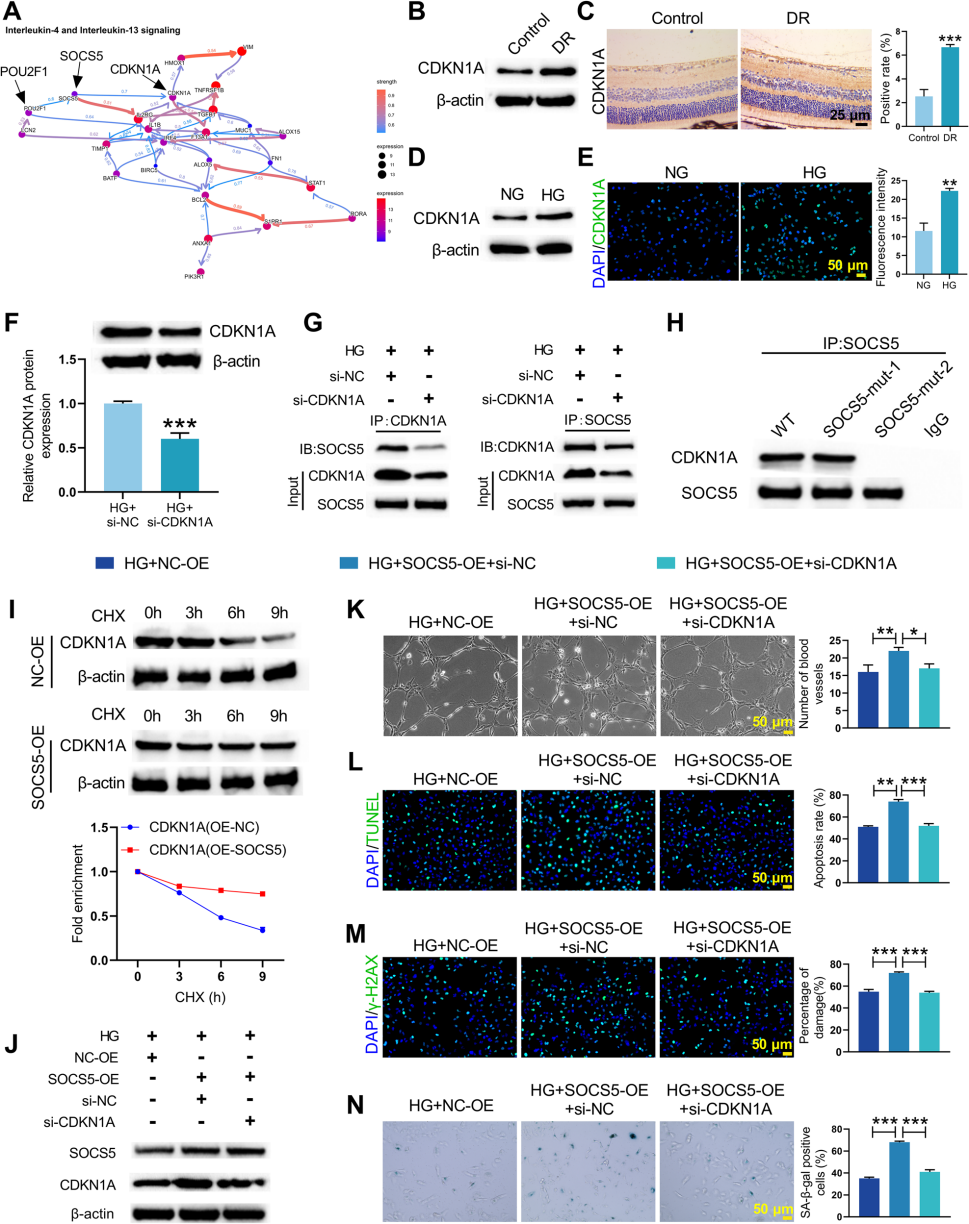
SOCS5 regulated DNA damage and senescence through stabilizing CDKN1A protein. **A** Potential SOCS5-related pathways and target genes were predicted by CBNplot. **B**, **C** WB and immunohistochemistry detection of CDKN1A expression in retinal tissues from control and DR mice. **D**, **E** CDKN1A expression in HRMECs treated with NG or HG was detected by WB and immunofluorescence. HRMECs were transfected with si-CDKN1A or si-NC under HG conditions. **F** CDKN1A protein levels in HRMECs were assessed by WB. **G** Co-IP assay demonstrating the interaction between CDKN1A and SOCS5 in HG-stimulated HRMECs. **H** Co-IP assay showing the interaction between CDKN1A and wild-type or domain-deletion mutants of SOCS5. **I** WB analysis of CDKN1A protein levels in HRMECs treated with or without SOCS5 overexpression at 0, 3, 6, and 9 h after treatment with CHX. HRMECs were co-transfected with SOCS5-OE and/or si-CDKN1A followed by HG exposure. **J** WB were employed to measure SOCS5 and CDKN1A expression in HRMECs undergoing genetic manipulation. **K** Matrigel tubule formation assay showing vascular formation in HRMECs after genetic manipulation. **L** TUNEL detection assessing apoptosis in HRMECs undergoing genetic manipulation. **M** γ-H2AX immunofluorescence assay was used to evaluate DNA damage in HRMECs after genetic manipulation. **N** SA-β-gal staining demonstrating senescence in HRMECs undergoing genetic manipulation. Student’s *t* tests were used for comparing two variables. A one-way ANOVA test was used for multiple variable comparison. Data are from n independent biological replicates (**B**, **C**, *n* = 3 mice; **D**–**N**, *n* = 3 independent cultures). *p ˂ 0.05, **p ˂ 0.01, ***p ˂ 0.001 vs Control/ NG/ HG+si-NC/ HG+si-SOCS5 + NC-OE.

In light of the established close involvement of inflammation in the onset and progression of DR, Reactome pathway enrichment analysis of preliminary sequencing data revealed significant enrichment of the “Interleukin-4 and Interleukin-13 signaling” pathway. To infer causal regulatory relationships within this pathway, CBNplot, an R package that reconstructs Bayesian networks from gene expression data to predict directional gene regulatory interactions [22], was employed. Application of this algorithm to the genes in the enriched interleukin signaling pathway indicated POU2F1 as the upstream regulator of SOCS5 and CDKN1A as the key downstream effector of SOCS5 (Fig. 4A). CDKN1A expression was elevated in DR mouse retinal tissues (Fig. 4B-C, Supplementary Fig. 3A, B) and in HG-stimulated HRMECs (Fig. 4D, E and Supplementary Fig. 3C, D). Subsequently, HRMECs were subjected to CDKN1A knockdown, and the knockdown efficiency was verified (Supplementary Fig. 1E, F). CDKN1A knockdown significantly reduced CDKN1A protein levels in HG-stimulated HRMECs, as confirmed by WB (Fig. 4F). Co-IP analysis revealed the interaction between CDKN1A and SOCS5 (Fig. 4G, H). The CHX chase assay demonstrated that SOCS5 overexpression significantly inhibited the degradation of CDKN1A protein (Fig. 4I). To investigate the functional relationship between CDKN1A and SOCS5, rescue experiments were performed. First, SOCS5 overexpression in HG-stimulated HRMECs elevated both SOCS5 and CDKN1A levels, and concurrent CDKN1A knockdown effectively reduced CDKN1A but not SOCS5 expression (Fig. 4J and Supplementary Fig. 3E, F). Functionally, SOCS5-OE exacerbated tubule formation, apoptosis, DNA damage, and cellular senescence, which were significantly rescued by si-CDKN1A (Fig. 4K–N). Conversely, SOCS5 knockdown reduced both SOCS5 and CDKN1A levels, while CDKN1A overexpression only restored CDKN1A expression (Supplementary Fig. 3G, H). Moreover, SOCS5 knockdown-induced reductions in tubule formation, apoptosis, DNA damage, and senescence were reversed by CDKN1A overexpression (Supplementary Fig. 3I–L). Together, these complementary gain- and loss-of-function studies establish CDKN1A as the essential downstream mediator through which SOCS5 exacerbates DNA damage and cellular senescence in DR progression.

### POU2F1 was upregulated in DR and promoted DNA damage and cellular senescence

**Fig. 5:**
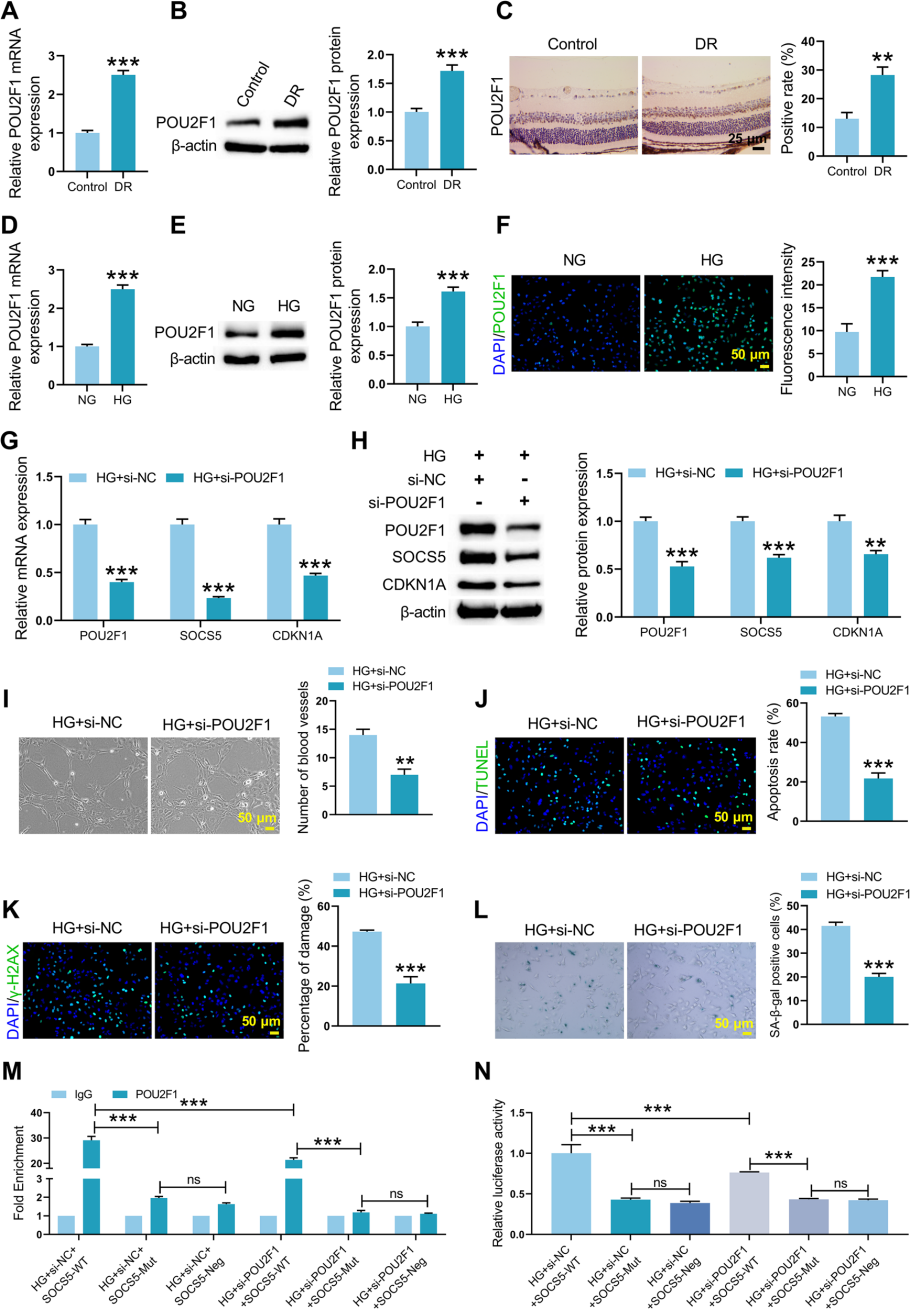
POU2F1 was upregulated in DR mice and regulated DNA damage and senescence in HG-treated HRMECs. **A**–**C** qRT-PCR, WB, and immunohistochemistry were performed to assess POU2F1 expression in retinal tissues from control and DR mice. **D**–**F** POU2F1 expression in HRMECs treated with NG or HG was detected by qRT-PCR, WB, and immunofluorescence. HRMECs were transfected with si-POU2F1 or si-NC, followed by HG stimulation. **G**, **H** The mRNA and protein levels of POU2F1, SOCS5, and CDKN1A in HRMECs with si-POU2F1 or si-NC were detected by qRT-PCR and WB. **I** Matrigel tubule formation assay assessing the vascular formation in HRMECs after si-POU2F1 or si-NC. **J** TUNEL detection evaluating apoptosis within HRMECs after si-POU2F1 or si-NC. **K** γ-H2AX immunofluorescence was used to detect intracellular DNA damage in HRMECs after si-POU2F1 or si-NC. **L** SA-β-gal staining analysis of cellular senescence in HRMECs after si-POU2F1 or si-NC. HG-stimulated HRMECs transfected with si-NC or si-POU2F1 were subjected to either the wild-type SOCS5 promoter (SOCS5-WT), a promoter with a mutated POU2F1 binding site (SOCS5-Mut), or a promoter region lacking the POU2F1 motif (SOCS5-Neg). **M** ChIP-qPCR analysis of POU2F1 binding to the SOCS5 promoter. **N** Dual-luciferase reporter assay measuring SOCS5 promoter activity. Student’s *t* tests were used for comparing two variables. Data are from n independent biological replicates (**A**–**C**, *n* = 3 mice; **D**–**N**, *n* = 3 independent cultures). *p ˂ 0.05, **p ˂ 0.01, ***p ˂ 0.001 vs Control/ NG/ HG+si-NC.

POU2F1 expression was increased in DR retinal tissues, as detected by qRT-PCR, WB and Immunohistochemical staining (Fig. 5A–C). In HG-treated HRMECs, POU2F1 levels were also upregulated than the NG group (Fig. 5D–F). To investigate the role of POU2F1, HRMECs were transfected with si-POU2F1. The transfection efficiency was validated (Supplementary Fig. 1F, G). qRT-PCR and WB analyses demonstrated significant downregulation of POU2F1, SOCS5, and CDKN1A in HG-treated HRMECs after POU2F1 knockdown (Fig. 5G, H). Furthermore, POU2F1 interference also reduced angiogenesis, apoptosis, DNA damage, and cellular senescence in HG-stimulated HRMECs (Fig. 5I–L). To establish the specificity of POU2F1 binding to the SOCS5 promoter, experiments were performed in HG-stimulated HRMECs transfected with si-NC or si-POU2F1, together with SOCS5-WT, SOCS5-Mut, or SOCS5-Neg. CHIP assay confirmed that POU2F1 was specifically enriched only at the SOCS5-WT fragment, with no significant enrichment detected at the SOCS5-Mut or SOCS5-Neg fragments. This specific enrichment was significantly attenuated by si-POU2F1 (Fig. 5M). Similarly, in dual-luciferase reporter assays, POU2F1 knockdown significantly reduced the activity of the SOCS5-WT promoter but not that of the SOCS5-Mut or SOCS5-Neg promoters (Fig. 5N). Therefore, POU2F1 promotes DNA damage and cellular senescence in DR and regulates the SOCS5-CDKN1A axis.

### POU2F1 promoted DNA damage and cellular senescence by regulating the transcriptional activation of SOCS5

**Fig. 6:**
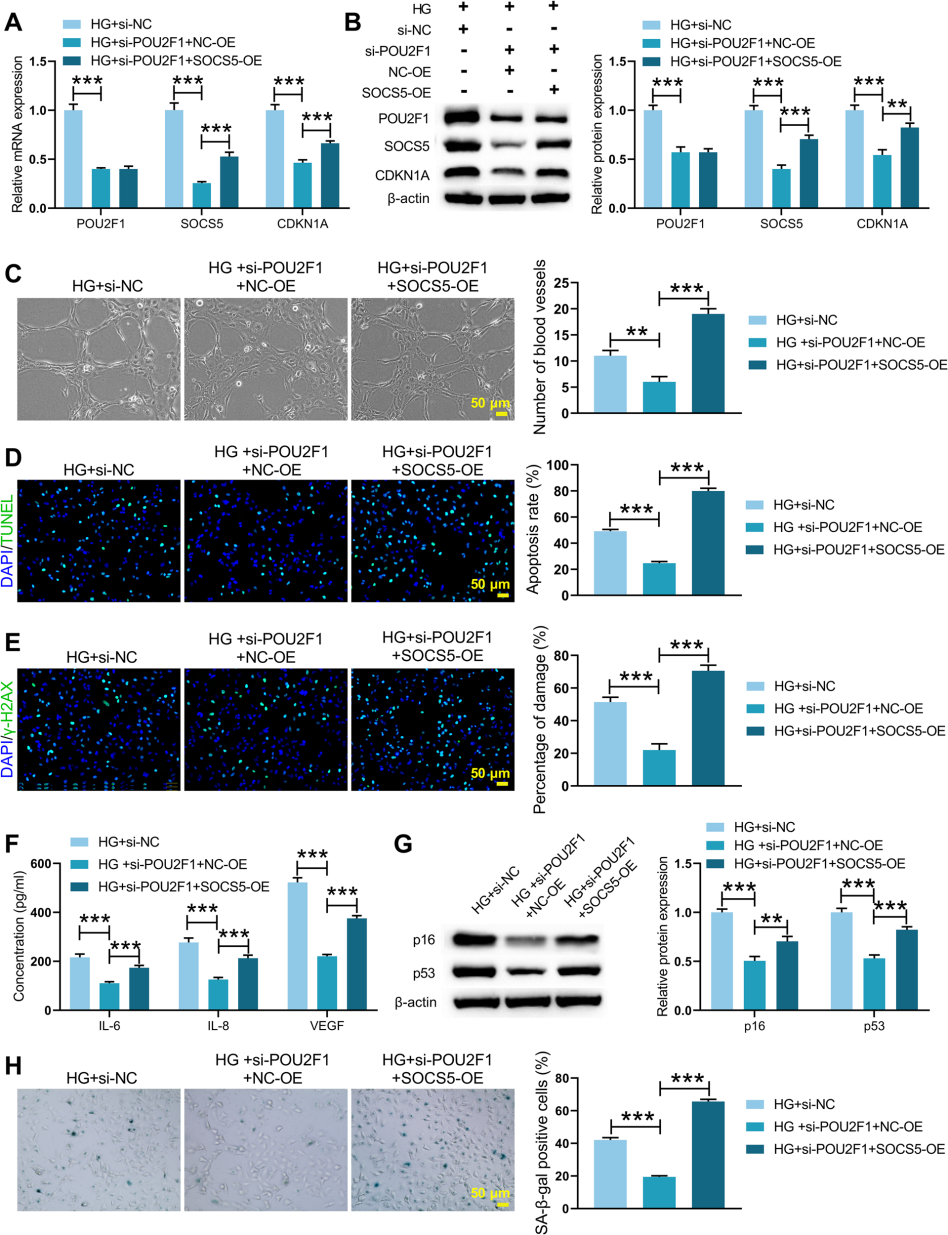
POU2F1 regulated DNA damage and senescence through SOCS5. HRMECs were co-transfected with si-POU2F1 and/or SOCS5-OE plasmids, followed by HG stimulation for 48 h. **A**, **B** The mRNA and protein levels of POU2F1, SOCS5, CDKN1A in genetically manipulated HRMECs were measured by qRT-PCR and WB. **C** Matrigel tubule formation assay assessing the vascular formation in genetically manipulated HRMECs. **D** TUNEL assay was performed to detect apoptosis in genetically manipulated HRMECs. **E** γ-H2AX immunofluorescence assay was used to evaluate DNA damage in genetically manipulated HRMECs. **F** ELISA analysis of senescence-associated secretory phenotypes IL-6, IL-8, and VEGF in genetically manipulated HRMECs. **G** WB was conducted to analyze the expression of senescence-related molecules p53 and p16 in genetically manipulated HRMECs. **H** SA-β-gal staining examining cell senescence in genetically manipulated HRMECs. A one-way ANOVA test was used for multiple variable comparison. Data are from n independent biological replicates (**A**–**H**, *n* = 3 independent cultures). *p ˂ 0.05, **p ˂ 0.01, ***p ˂ 0.001 vs HG+si-NC/ HG+si-POU2F1 + NC-OE.

To determine whether POU2F1 mediates DNA damage and cellular senescence through SOCS5, HRMECs were treated with si-POU2F1 and/or SOCS5-OE followed by HG induction. POU2F1 knockdown reduced the expression of POU2F1, SOCS5, and CDKN1A, while SOCS5 overexpression increased SOCS5 and CDKN1A levels but not POU2F1 (Fig. 6A, B). Similarly, si-POU2F1 reduced the number of tubule formations, apoptosis, and DNA damage in HG-induced HRMECs, while SOCS5-OE reversed this phenomenon (Fig. 6C–E and Supplementary Fig. 4). In addition, SOCS5 overexpression also restored the levels of IL-6, IL-8, VEGF, p53 and p16 in HG-induced HRMECs that were suppressed by si‑POU2F1 (Fig. 6F, G). SA-β-gal further confirmed that si-POU2F1 inhibited cell senescence in HG-induced HRMECs, which was counteracted by SOCS5-OE (Fig. 6H). Thus, POU2F1 regulates DNA damage and senescence through transcriptional activation of SOCS5.

### POU2F1 knockdown inhibited DNA damage and cellular senescence to ameliorate DR in vivo

**Fig. 7:**
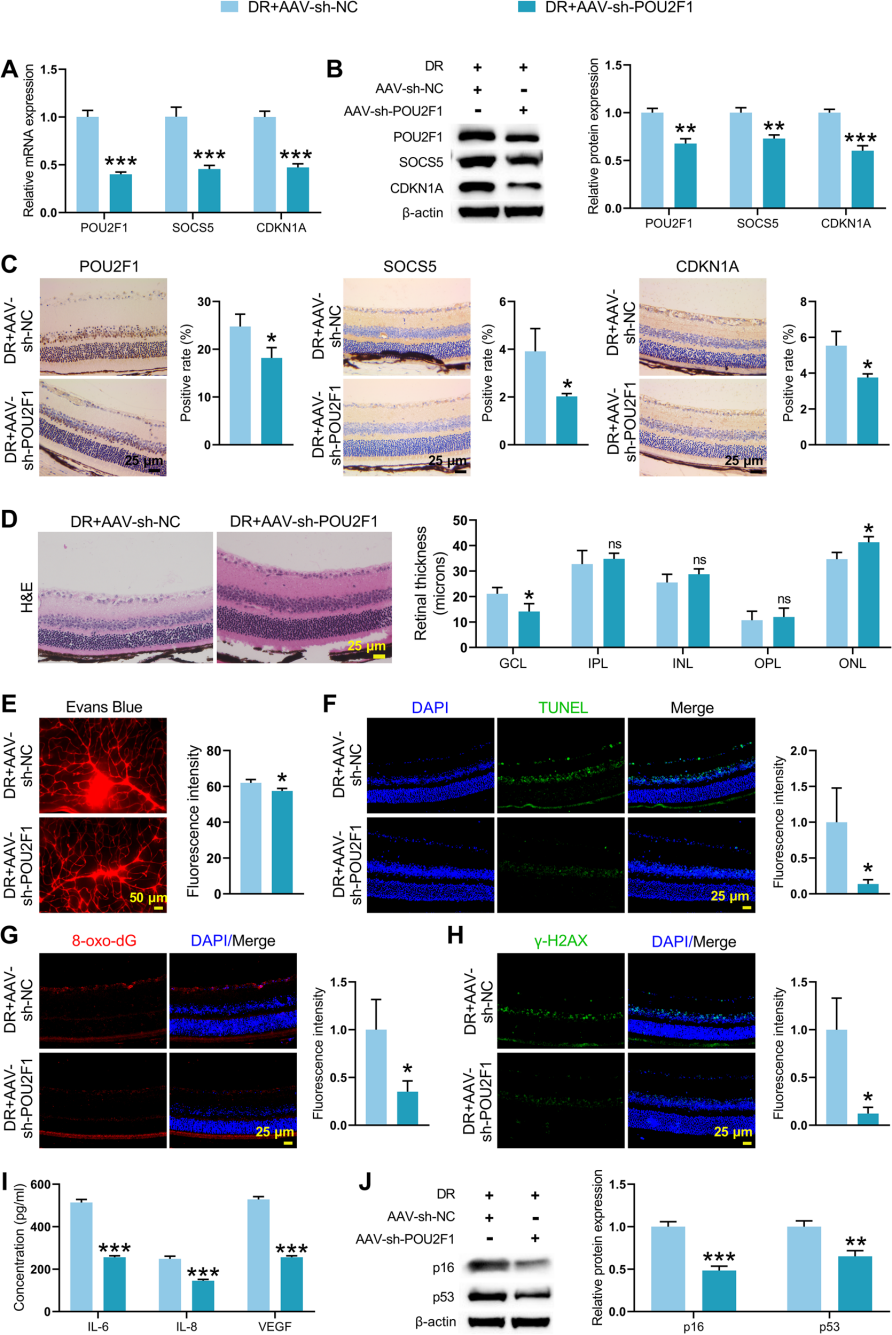
Knockdown of POU2F1 altered DNA damage and senescence in retinal tissues of DR mice. STZ-induced DR mice were intravitreally injected with AAV-sh-NC or AAV-sh-POU2F1 (*n* = 9 mice per group). **A**–**C** qRT-PCR, WB and immunohistochemistry were used to detect POU2F1, SOCS5, and CDKN1A expression in retinal tissues from DR mice with or without sh-POU2F1. **D** H&E staining of retinal sections showing structural pathological changes in DR mice with or without sh-POU2F1. **E** Evans Blue staining was performed to observe retinal vasculature in DR mice with or without sh-POU2F1. **F** TUNEL staining assessing retinal tissue apoptosis in DR mice with or without sh-POU2F1. **G**, **H** Evaluation of oxidative DNA damage (8-oxo-dG) and DNA damage (γ-H2AX) was conducted by immunofluorescence staining in retinal tissues from DR mice with or without sh-POU2F1. **I** ELISA analysis of senescence-related secretory phenotypes IL-6, IL-8, and VEGF in retinal tissues from DR mice with or without sh-POU2F1. **J** WB was performed to detect the expression of retinal tissue senescence-related molecules p53 and p16 in retinal tissues from DR mice with or without sh-POU2F1. Student’s *t* tests were used for comparing two variables. Data are from n independent biological replicates (**A**–**J**, *n* = 3 mice). *p ˂ 0.05, **p ˂ 0.01, ***p ˂ 0.001 vs DR + AAV-sh-NC. ns means no significant difference vs DR + AAV-sh-NC group.

To validate the in vivo effect of POU2F1, AAV-sh-POU2F1 was intravitreally injected to knock down POU2F1, and the knockdown efficiency was verified (Supplementary Fig. 1I, J). POU2F1, SOCS5, and CDKN1A expression was significantly reduced in retinal tissues of POU2F1‑knockdown DR mice (Fig. 7A, B). Histopathological analyses confirmed that knockdown of POU2F1 ameliorated retinal pathology in mice, as evidenced by reduced swelling of the retinal ganglion cell layer, more regular cellular arrangement of the inner and outer nuclear layers, and decreased thickening of the inner plexiform layer in DR mice (Fig. 7C, D). Evans Blue staining and TUNEL assay showed that POU2F1 knockdown improved vascular integrity and reduced apoptosis in retinal tissues from DR mice (Fig. 7E, F). Additionally, 8-oxo-dG and γ-H2AX immunofluorescence indicated reduced retinal DNA damage in retinas of POU2F1‑knockdown DR mice (Fig. 7G, H). ELISA and WB assays revealed that sh-POU2F1 reduced the levels of IL-6, IL-8, VEGF, p16 and p53 in DR mouse retinal tissues (Fig. 7I, J). Collectively, these results indicate that POU2F1 knockdown alleviates DR progression by attenuating DNA damage and cellular senescence in retinal tissues. Analysis of clinical samples showed elevated protein levels of CDKN1A, SOCS5, and POU2F1 proteins in the peripheral blood of DR patients compared to healthy controls (Supplementary Fig. 5), suggesting the clinical relevance of the POU2F1-SOCS5-CDKN1A axis.

## Discussion

As a serious complication of diabetes mellitus, the pathogenesis of DR is complex and involves many factors [23]. Although recent advances have deepened our understanding of DR [24], the specific molecular mechanisms driving its progression remain incompletely elucidated. In this study, we systematically probed into the role of SOCS5 in DR development using in vivo and in vitro models combined with multi‑method experimental approaches. Our findings confirm SOCS5 as a key gene in DR and delineate a novel mechanism through which it promotes disease progression by regulating DNA damage and cellular senescence. These discoveries offer novel insights into the pathology of DR and contribute to the identification of effective therapeutic targets.

SOCS proteins have been identified as key negative regulators of various cytokines, modulating the JAK/STAT signaling pathway and thereby regulating genes involved in cell differentiation, proliferation, inflammation, and fibrosis [25, 26]. While SOCS proteins are known to contribute to diabetes development [27–29], the role of SOCS5 in the progression of diabetes-related complications remains poorly understood compared to SOCS1 and SOCS3. Previous studies have shown aberrantly elevated expression of SOCS5 in STZ-induced diabetic mice [30]. Similarly, this study found that SOCS5 was significantly upregulated in both DR mice and HG-induced HRMECs. Notably, Angpt2-mediated upregulation of SOCS5 has been reported to enhance mesangial cell apoptosis by inhibiting the JAK1/STAT3 pathway, thereby aggravating renal dysfunction in diabetic nephropathy [31]. This study innovatively revealed that upregulation of SOCS5 in a DR model promoted DNA damage and cellular senescence, leading to retinal histopathological injury, highlighting the context-dependent and tissue-specific roles of SOCS5 under different pathological conditions.

CDKN1A plays a critical role in the DNA damage response, cell cycle arrest and cellular senescence [32, 33]. It has been demonstrated that CDKN1A is upregulated in diabetic nephropathy and diabetic cognitive impairment, and inhibition of CDKN1A expression can ameliorate senescence in renal tubular and cerebrovascular cells [34–36]. Here, we further confirmed the upregulation of CDKN1A in the context of DR and its contribution to DNA damage and cellular senescence in HRMECs. Moreover, through CBNplot analysis, CDKN1A was predicted to be a downstream target of SOCS5 in DR. Co-IP and CHX chase assays verified that SOCS5 directly regulated the protein stability of CDKN1A. Subsequent rescue experiments further established that SOCS5 modulated DNA damage and cellular senescence in DR by regulating CDKN1A. These results innovatively revealed the critical role of the SOCS5-CDKN1A axis in the pathogenesis of DR.

Another novel finding is that POU2F1 promotes SOCS5 transcription, thereby contributing to retinal tissue injury. As a widely expressed transcription factor, POU2F1 plays a key role in regulating inflammation- and cell cycle-related genes and has been implicated in susceptibility to type 2 diabetes [37, 38]. The present study first demonstrated POU2F1 upregulation in DR and its role in DR progression via modulating the expression of SOCS5. Recent studies have reported that downregulation of POU2F1 promotes DNA damage and growth inhibition in colorectal cancer cells [39]. A retrospective clinical study also suggests that high POU2F1 expression is associated with improved survival in smokers with lung adenocarcinoma and inhibits clonogenic growth and proliferation in A549 lung cancer cells [40]. Although these observations appear contradictory to our findings that POU2F1 exacerbates retinal damage in DR by enhancing DNA damage and senescence through the SOCS5-CDKN1A axis, the discrepancy likely reflects fundamental differences between carcinogenesis and diabetic microvascular complications. The previously unrecognized role of POU2F1 in promoting DNA damage and senescence in retinal endothelial cells, as revealed in this study, contrasts with its anti-proliferative function in oncology, thereby emphasizing both the novelty and context-dependency of the pathogenic pathway we propose in DR.

Despite the important findings of this study, some limitations remain. The C57BL/6 mouse model was primarily used to study DR in this study. Although this model is widely employed in diabetes research, its pathophysiological processes still differ from those of human diabetic retinopathy. Therefore, validation in more human-relevant models is necessary. Recent advances in human induced pluripotent stem cell-derived retinal organoids offer a promising path in this direction [41, 42]. These models recapitulate key features of DR in a human-specific context, including neurodegeneration, inflammation, and microvascular dysfunction [41, 42]. Future studies using such organoids may help validate the proposed mechanism in biologically relevant human tissue and explore the potential of SOCS5 as a biomarker or therapeutic target. In summary, this study revealed a novel POU2F1-SOCS5-CDKN1A axis involved in retinal DNA damage and senescence in DR, providing new insight and approaches for the clinical treatment of DR.

## Conclusion

This study reveals the molecular mechanism by which SOCS5 promotes the development of DR by regulating CDKN1A-mediated DNA damage and cellular senescence and clarifies the role of POU2F1 as an upstream transcriptional regulator. Targeted inhibition of SOCS5 or POU2F1 can alleviate the pathological process of DR, providing a theoretical basis for the development of novel therapeutic strategies.

## Materials and methods

### Bioinformatics analysis

A bioinformatics analysis was performed on transcriptomic data from whole blood samples categorized into four clinical groups: Normal (NC, *n* = 15), Diabetes mellitus (DM, *n* = 16), Non-proliferative diabetic retinopathy (NPDR, *n* = 15), and Proliferative diabetic retinopathy (PDR, *n* = 15). Trend analysis was conducted using the Mfuzz package to identify genes whose expression followed the Normal→DM → NPDR → PDR progression pattern. Weighted Gene Co-expression Network Analysis (WGCNA) was then employed to identify gene modules significantly correlated with disease progression. Candidate genes were further refined using machine learning approaches, including Least Absolute Shrinkage and Selection Operator (LASSO) regression and Support Vector Machine-Recursive Feature Elimination (SVM-RFE). The intersection of genes identified by both methods yielded four key candidates, among which SOCS5 was selected for experimental validation.

### Clinical samples

Peripheral blood samples were collected from non-diabetic control individuals and patients with DR after obtaining written informed consent from all participants. All participants signed the informed consent. The protocols conformed to the Declaration of Helsinki and were approved by the ethical review committee of the First Affiliated Hospital of Kunming Medical University.

### Animals

Eight-week-old C57BL/6J mice (purchased from Beijing SPF Biotechnology Co., Ltd.) were housed in a specific pathogen-free (SPF) standard environment with controlled temperature (21–26 °C) and humidity (30–70%). The sample size in each group was determined using the Resource Equation Method, aiming to maintain the error degrees of freedom (DF) in an analysis of variance (ANOVA) within the acceptable range of 10 to 20. For the two-group comparisons planned in this study, this method yielded a recommended sample size range of 6–11 animals per group. A sample size of nine mice per group was chosen to ensure sufficient statistical power while adhering to the 3 R (Replacement, Reduction, Refinement) principles. A post-hoc power analysis using G*Power 3.1 on a key outcome (area of pathological neovascularization) confirmed high power (0.96) with this sample size, given the observed effect size (Cohen’s d = 1.45). After one week of acclimatization, the experiments were conducted. The Ethics Committee of Kunming Medical University approved all study protocols (Approval No: Kmmu20240605).

### Construction of DR mouse models

C57BL/6J mice were randomly divided into two groups: the Control group and the DR group. After 4 weeks of high‑fat diet feeding, the DR group received intraperitoneal injections of streptozotocin (STZ) (at a dose of 60 mg/kg) for 5 consecutive days. One week after the injection, fasting blood glucose levels were measured by tail-tip blood collection, and a blood glucose value of 16.7 mmol/L or more was used as a criterion for successful modeling. All mice included in this study were successfully modeled, and no exclusions were necessary. On the second day after blood glucose measurement, 2 μL of adeno-associated virus and control virus (with serological type 2 of the virus) were injected into the vitreous cavity. After 12 weeks of feeding, to maximize data collection from each animal and minimize total usage, the mice in each group were allocated to different endpoint analyses: three mice in each group were selected for tail vein injection of Evans blue to assess the vascular leakage in retinal tiling; the retinal tissues of three mice in each group were frozen and preserved; and the eyes of the remaining three mice in each group were fixed with tissue fixative and prepared as paraffin-embedded wax blocks for subsequent analyses. All subsequent histological, molecular, and biochemical analyses were performed by investigators blinded to the group assignments.

### Cell culture and treatment

Human retinal microvascular endothelial cells (HRMECs) (BFN60804011, Shanghai Cell Bank) were cultured in an endothelial cell-specific medium at 37 °C under a humidified atmosphere containing 5% CO₂. The identity of the cell line was authenticated by short tandem repeat (STR) profiling, and the verification report is provided in the Supplementary Materials (File 1).

To establish the in vitro DR model, HRMECs were stimulated with 50 mmol/L HG. Control cells were cultured in normal glucose (NG, 5.5 mmol/L) supplemented with 50 mmol/L mannitol to achieve the same osmolality. For investigating the functional interactions among POU2F1, SOCS5, and CDKN1A, HRMECs were transfected with plasmids or siRNAs targeting POU2F1, SOCS5, and/or CDKN1A, followed by HG stimulation or appropriate control treatments. Cells were harvested 48 h post‑transfection for subsequent analyses.

### Quantitative real-time fluorescence PCR (qRT-PCR)

Total RNA from cells or tissues was extracted using the TRIzol reagent (G3013, Servicebio). Total RNA was reverse transcribed into cDNA with PrimeScript RT reagent Kit (RR037Q, Takara). Gene expression levels of POU2F1, SOCS5, and CDKN1A were quantified using GAPDH as the internal control. Each reaction was run in triplicate, and relative expression was calculated via the 2^-∆∆CT^ method. The primers used in qRT-PCR are listed in Table 1.

**Table 1:** Primers of qRT-PCR and CHIP-qPCR.

Gene	Forward sequence (5’-3’)	Reverse sequence (5’-3’)
GAPDH (Human/Mouse)	GCACCGTCAAGGCTGAGAAC	TGGTGAAGACGCCAGTGGA
POU2F1 (Human)	ATGAACAATCCGTCAGAAACCAG	GATGGAGATGTCCAAGGAAAGC
SOCS5 (Human)	GTGCCACAGAAATCCCTCAAA	TCTCTTCGTGCAAGTCTTGTTC
CDKN1A (Human)	TGTCCGTCAGAACCCATGC	AAAGTCGAAGTTCCATCGCTC
POU2F1 (Mouse)	AGCTGGGACAAGTTTACAGGC	TCCCGACTCTTCACTGGATTTA
SOCS5 (Mouse)	GAACCCCAACAGATGTCCGTC	GGATCTCTGCGGCACAGTTTT
CDKN1A (Mouse)	CCTGGTGATGTCCGACCTG	CCATGAGCGCATCGCAATC

### Western blotting (WB)

Retinal tissues or cells were lysed using RIPA lysis buffer (BL504A, Bio-sharp), followed by protein quantification using the Pierce BCA protein quantification kit (BL521A, Bio-sharp). Proteins were separated using 10% SDS-PAGE and transferred to PVDF membranes (A1030, Solarbio) using wet transfer. Membranes were blocked with 5% skim milk for 2 h at room temperature. Antibodies were diluted to the target concentration in the sealing solution and incubated with the membrane at 4 °C overnight. Antibodies used included: SOCS5 Primary Antibody (1:1000, A05989-1, BOSTER), p53 Primary Antibody (1:1000, ab26, Abcam), p16 Primary Antibody (1:1000, ab51243, Abcam), CDKN1A Primary Antibody (1:1000, BM3990, BOSTER) and POU2F1 primary antibody (1:1000, PB9317, BOSTER). After washing, membranes were incubated with HRP‑conjugated secondary antibodies (anti‑mouse IgG, 1:5000, ab6789; anti‑rabbit IgG, 1:5000, ab6721; both from Abcam) at 37 °C for 2 h. Signals were detected using an all-in-one chemiluminescent imaging system (ChampChemi 910, SINSAGE).

### Evans blue assay

For each group, three mice were randomly anesthetized. After enucleation, 200 µL of Evans blue dye (R28420, Genye Bio) was administered through the tail vein. Mouse eyeballs were then collected and fixed in standard tissue fixative for 45 min. Retinal flat‑mounts were prepared and imaged under a fluorescence microscope.

### Histopathological analysis

Mouse retinal tissue samples were fixed using 4% paraformaldehyde, dehydrated, cleared, and embedded in paraffin. Sections were stained with hematoxylin and eosin (H&E) and examined under a microscope for observation and photography. The histological changes were quantitatively scored according to the Suzuki scoring criteria.

### Immunofluorescence staining

HRMECs cells were fixed with 4% paraformaldehyde for 30 min, permeabilized with 0.1% Triton X‑100 for 10 min, and blocked with 5% bovine serum albumin for 1 h at room temperature. Cells were incubated overnight at 4 °C with primary antibodies against DRP1, COXIV, γ‑H2AX, or 8‑oxoG (4354-MC-050, Bio-Techne). After incubation, slides were incubated with appropriate secondary antibodies and counterstained with DAPI. Finally, images were acquired with the aid of a fluorescence microscope (BZ-X800, Keens).

### Matrigel tubule formation

Matrigel matrix was thawed overnight at 4 °C, mixed gently, and pipetted into a confocal Petri dish (800 µL/dish, FCFC020-10pcs, BeyoGold). After polymerization at 37 °C for 1 h, HRMECs were trypsinized, counted, and resuspended in serum‑free DMEM at 2 × 10⁵cells/mL. 1 mL of cells was added to the petri dish. After incubation at 37 °C for 3 h, the tubule formation was observed and photographed using a microscope (BZ-X800, Keens).

### Terminal deoxynucleotidyl transferase dUTP nick end labeling (TUNEL) staining

Apoptosis was assessed using the TUNEL Apoptosis Detection Kit (C1086, Beyotime). Briefly, cells were fixed with 4% paraformaldehyde for 30 min and permeabilized with 0.3% Triton X‑100 in PBS for 5 min at room temperature. After washing, cells were incubated with 100 µL TUNEL reaction mixture at 37 °C for 60 min in the dark. Nuclei were counterstained with a DAPI solution. Finally, images were collected by fluorescence microscopy.

### Senescence-associated β-galactosidase staining

Cellular senescence was evaluated with the senescence-associated β-galactosidase staining kit (C0602, Beyotime). HRMECs were seeded in 48‑well plates and cultured until 70–80% confluent. Cells were fixed with β‑galactosidase staining fixative for 15 min at room temperature, washed, and incubated with staining working solution overnight at 37 °C. Finally, the plates were observed and photographed with a fluorescence microscope.

### Enzyme-linked immunosorbent assay (ELISA)

The levels of senescence‑associated secretory phenotype factors (IL‑6, IL‑8, and VEGF) were quantitatively analyzed by ELISA kits (E-EL-H6156 for IL‑6, E-EL-H6008 for IL‑8, and E-EL-H0111 for VEGF; Elabscience) according to the manufacturer’s protocols. The absorbance was measured at 405 nm by Multiskan FC (Thermo Fisher).

### Co-immunoprecipitation (Co-IP)

Cells were lysed in RIPA lysate (89900, Thermo Fisher). Primary antibodies (POU2F1 or SOC5S) were incubated with Protein A + G magnetic beads for 2 h according to the instructions of the immunoprecipitation kit (P2179S, Beyotime). Subsequently, the cell lysates were incubated with the primary antibody-magnetic bead complexes overnight at 4 °C on a shaker at a constant temperature. On the following day, the magnetic bead complexes containing protein lysates were collected by centrifugation. Finally, the immunoprecipitates were analyzed by protein immunoblotting.

To map the SOCS5‑CDKN1A interaction domain, Co‑IP was performed in HRMECs transfected with wild‑type (SOCS5‑WT) or domain‑deletion mutants (SOCS5‑mut‑1, SOCS5‑mut‑2). Primer sequences for SOCS5 constructs are listed: SOCS5-WT (5′-CGTGATCGGGGTCGTGGTGGG-3′), SOCS5-mut-1 (5′-CGTGATCGGTGTCGTGGTGGG-3′), and SOCS5-mut-2 (5′-CGTGATCGGGGTTGTGGTGGG-3′). Transfected cells were lysed and subjected to Co‑IP as described above.

### Cycloheximide (CHX) chase assay

To investigate the effect of SOCS5 on the protein stability of CDKN1A, a CHX chase assay was performed. HRMECs were transfected with either OE-NC or OE-SOCS5 and cultured for 20 h. The culture medium was then replaced with fresh medium containing 2–3 μg/mL CHX (2112, Cell Signaling) to inhibit de novo protein synthesis. Cells were harvested at 0, 3, 6, and 9 h after CHX treatment, washed twice with PBS, and lysed using RIPA buffer supplemented with protease inhibitors. Proteins extracted from the lysed cells were subjected to WB analysis and protein quantification using the methods described above.

### Chromatin immunoprecipitation (CHIP)-qPCR

According to the manufacturer’s instructions, the CHIP assay was performed using the CHIP kit (P2078, Beyotime). Briefly, HRMECs cells were fixed with 1% formaldehyde at room temperature for 10 min. The cells were then collected and lysed, followed by chromatin fragmentation through sonication. Immunoprecipitation was carried out with antibodies against POU2F1 or control IgG, followed by incubation with Protein A/G magnetic beads. After washing, bound DNA was eluted, purified, and analyzed by qPCR. The primer sequences used in the CHIP assay are listed in Table 1.

### Dual-luciferase reporter assay

To investigate the transcriptional regulation of SOCS5 by POU2F1, dual-luciferase reporter assays were performed. A fragment of the human SOCS5 promoter was cloned into the pGL3-Basic vector to generate the SOCS5 promoter-driven firefly luciferase reporter plasmid. Three luciferase reporter constructs were generated: a wild‑type SOCS5 promoter reporter (SOCS5‑WT), a promoter with a mutated POU2F1‑binding site (SOCS5‑Mut), and a promoter fragment lacking the POU2F1 motif (SOCS5‑Neg). HRMECs (70–80% confluent) in 6‑well plates were co‑transfected with each reporter plasmid, the Renilla luciferase control plasmid, and either si‑POU2F1 or control siRNA. All transfections were performed using Lipo8000™ transfection reagent (C0533, Beyotime) according to the manufacturer’s instructions. After 48 h, luciferase activity was measured using a dual-luciferase reporter assay kit (RG027, Beyotime) on a TECAN Spark multimode microplate reader.

### Blinded image acquisition and quantification

To ensure objective assessment, all histological and immunofluorescence staining images were acquired and quantified in a blinded manner. After acquisition, images were assigned random codes by an experimenter not involved in the subsequent analysis. The coded images were then provided to an independent researcher who performed all quantitative measurements using ImageJ software without knowledge of group assignments. The quantification data were recorded and matched to the experimental groups only after analysis was complete.

### Statistical analysis

Statistical analyses were conducted on data derived from three independent biological replicates, with three technical replicates used for each measurement within every biological replicate sample. Statistical analyses were performed using GraphPad Prism 8.0. The normality of data distribution was assessed using the Shapiro-Wilk test. For comparisons between two groups with normal distribution and homogeneity of variance, an unpaired Student’s *t* test was applied; in cases of violated variance homogeneity, Welch’s t-test was used. For comparisons among multiple groups, one-way ANOVA was performed when normality and homogeneity of variance assumptions were met. If the variance was uneven, the Brown-Forsythe correction was applied. For data that did not follow a normal distribution, the Mann-Whitney U test was used for two-group comparisons, and the Kruskal-Wallis test was used for multiple groups. Homogeneity of variance was evaluated using Levene’s test. A *p*-value of less than 0.05 was considered statistically significant.

### Ethics statement

The conduct of this research adheres to the ethical standards set forth by the Laboratory Animal Center of The First Affiliated Hospital of Kunming Medical University (Kmmu20240605). All animal studies adhered to the guidelines established by the institution’s ethical committee. All participants signed the informed consent. The protocols conformed to the Declaration of Helsinki and were approved by the ethical review committee of the First Affiliated Hospital of Kunming Medical University.

## Supplementary information

Original Western blots

Supplementary figures

File 1-STR-HRCEC

aj-checklist

## Data Availability

All data related to the article can be obtained from the corresponding author upon reasonable request.
