# Supplementary material for: Mechanistic insights into SOCS5-related DNA damage and cellular senescence in diabetic retinopathy
Source: Cell Death Discov. 2026 Apr 1;12:212. doi: 10.1038/s41420-026-03011-3 (PMC13168495; doi:10.1038/s41420-026-03011-3)

Figure 2D-SOCS5


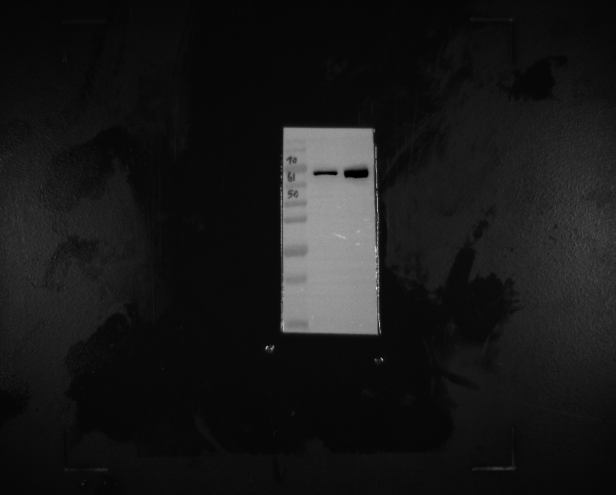


Figure 2D-β-actin


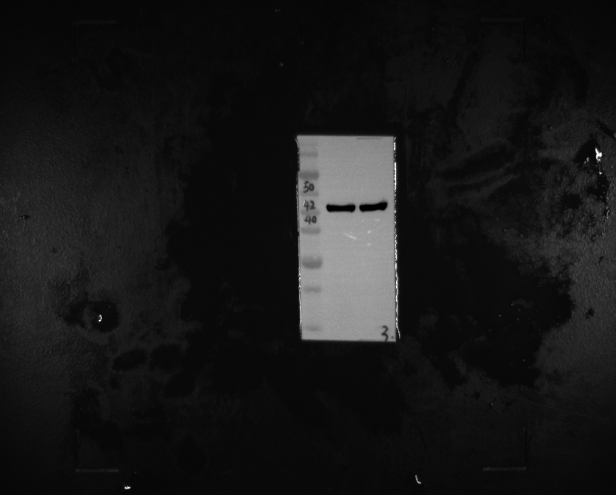


Figure 2G-SOCS5


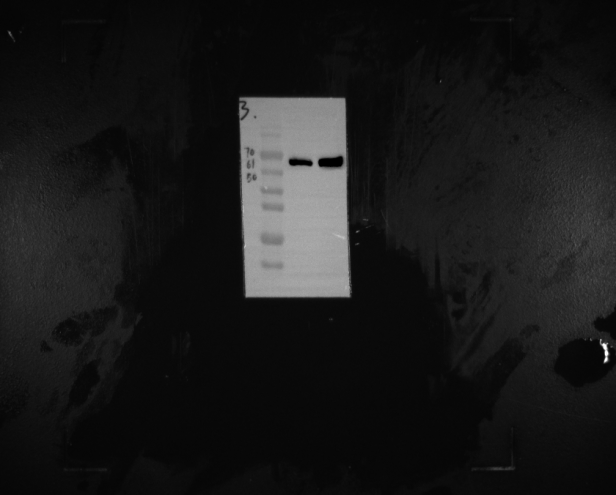


Figure 2G-β-actin


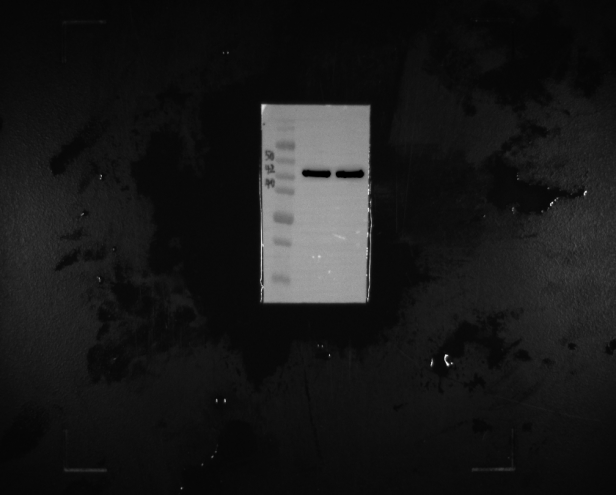


Figure 3E-P16


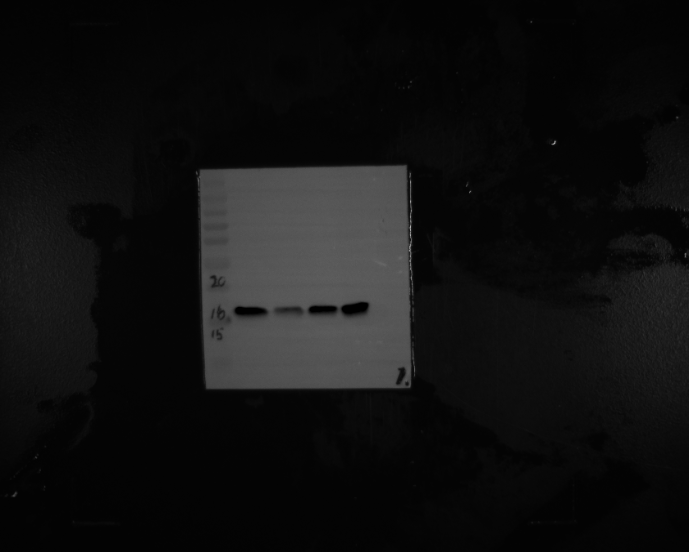


Figure 3E-P53


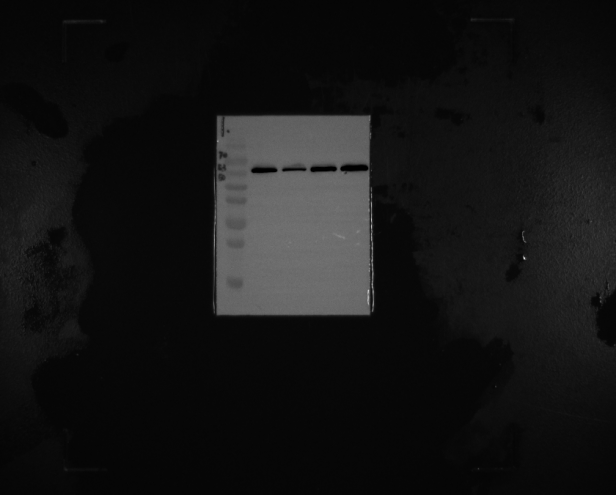


Figure 3E-β-actin


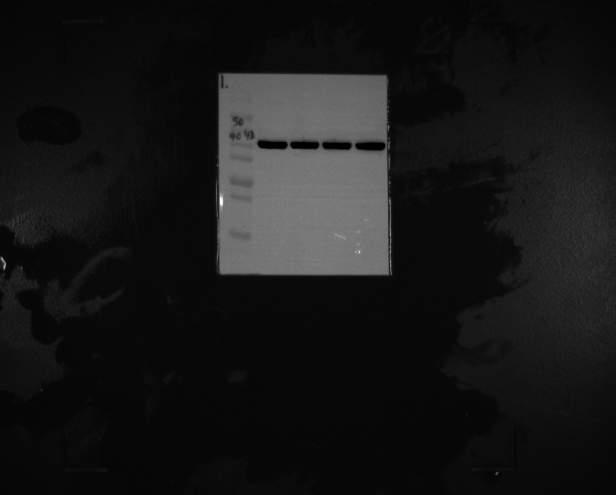


Figure 3K-P16

**
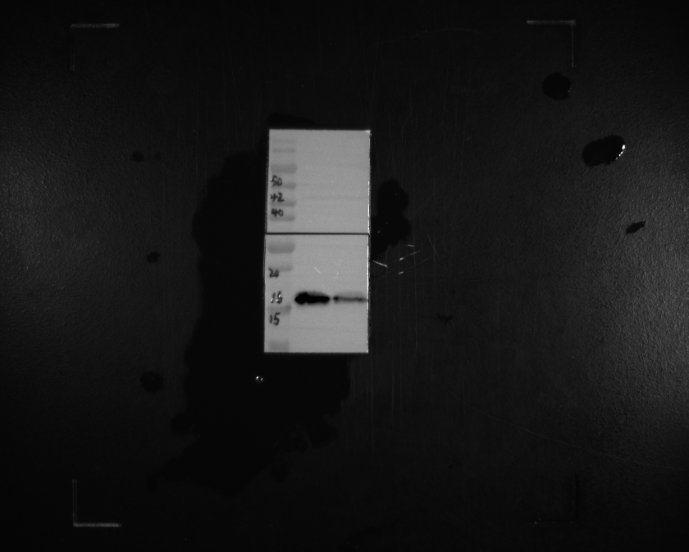
**

Figure 3K-β-actin(P16)


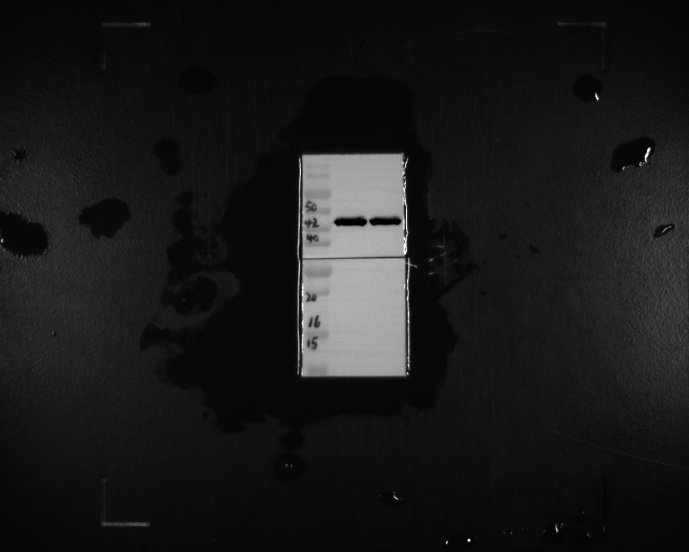


Figure 3K-P53


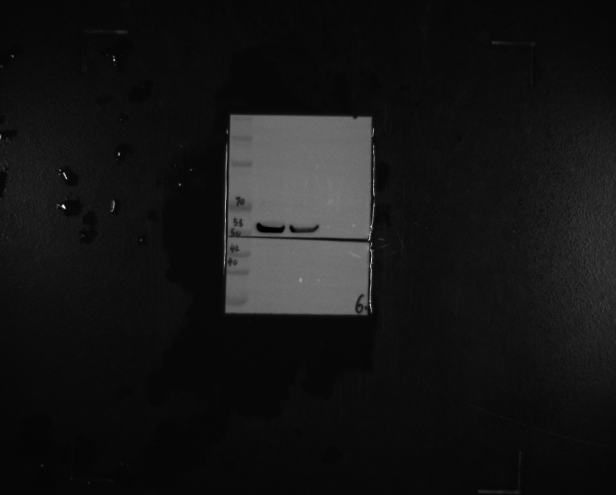


Figure 3K-β-actin(P53)


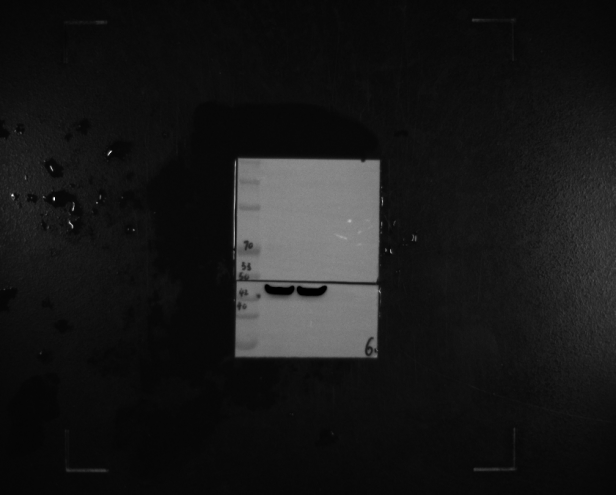


Figure 4B-CDKN1A


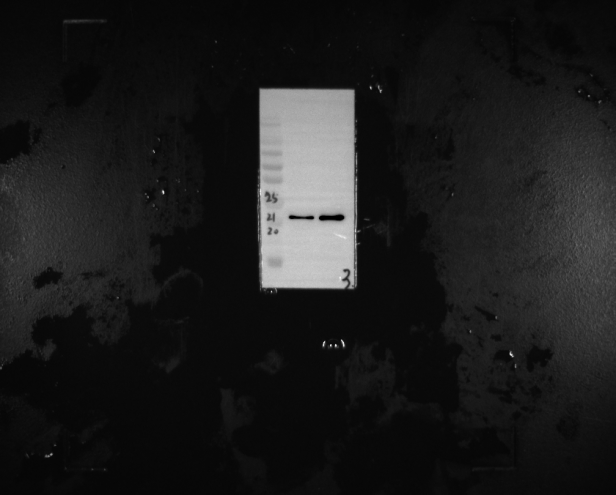


Figure 4B**-**β-actin


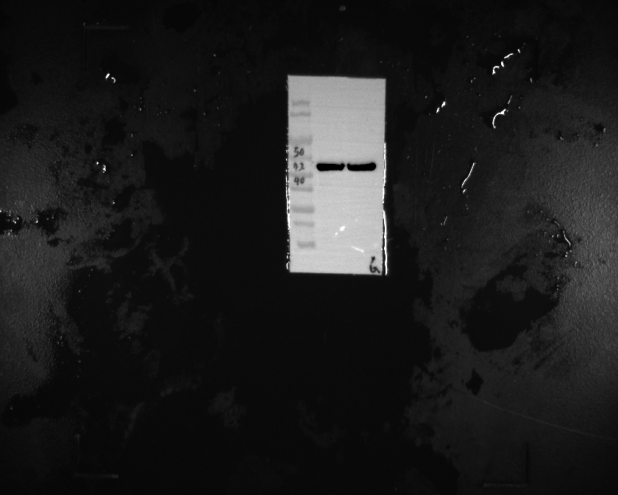


Figure 4D-CDKN1A


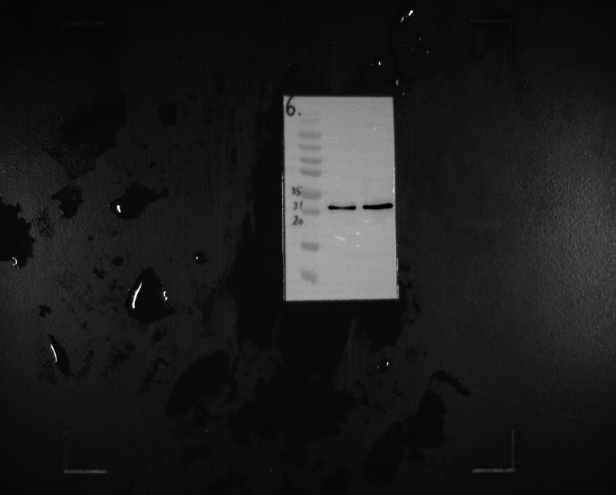


Figure 4D**-**β-actin

**
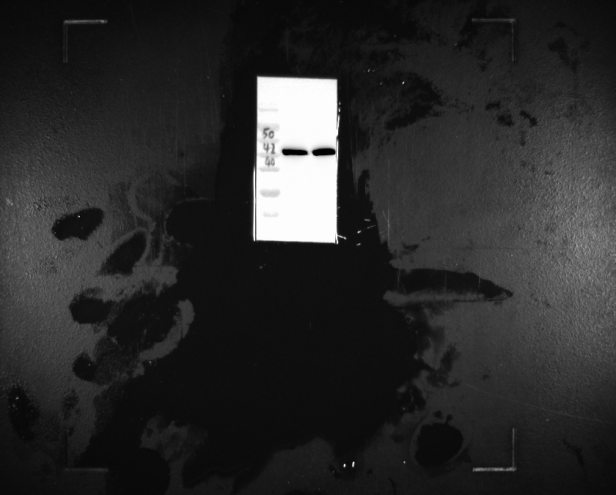
**

Figure 4F-CDKN1A


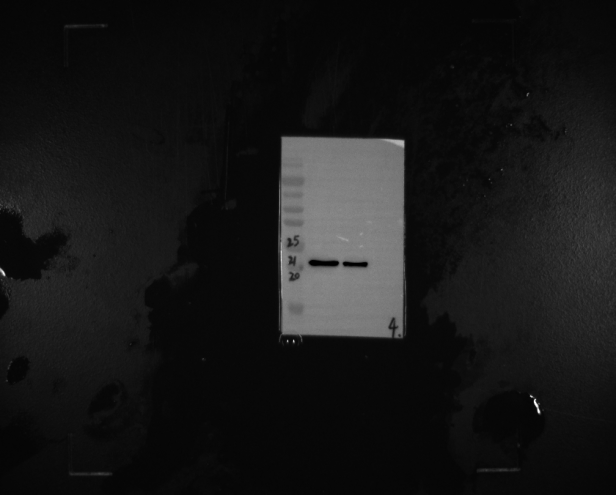


Figure 4F**-**β-actin


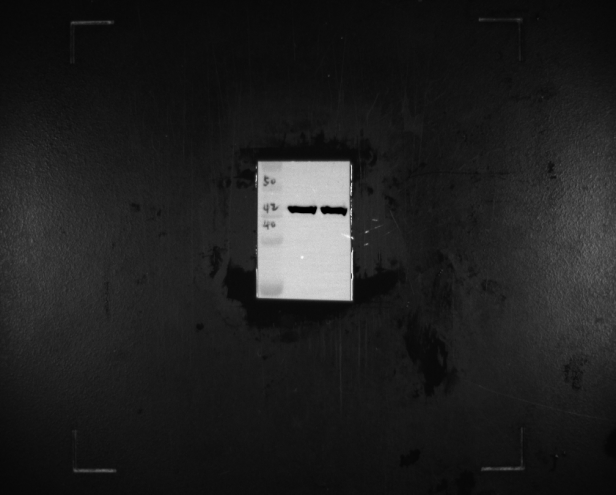


Figure 4G-CDKN1A


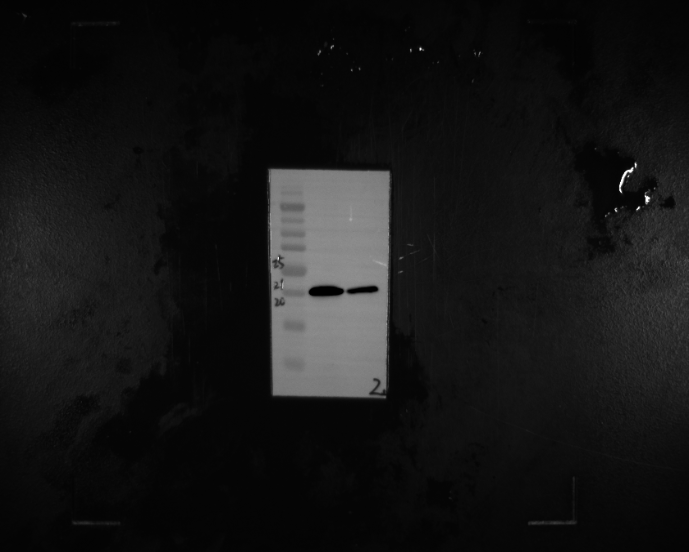


Figure 4G-IB-SOCS5


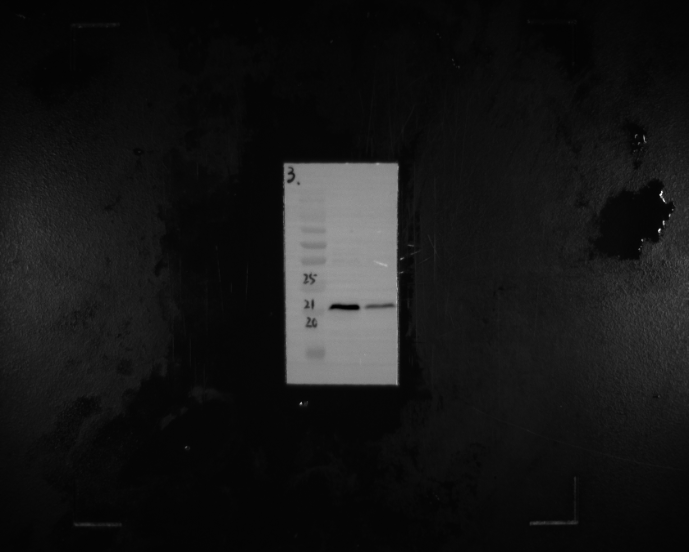


Figure 4G-SOCS5


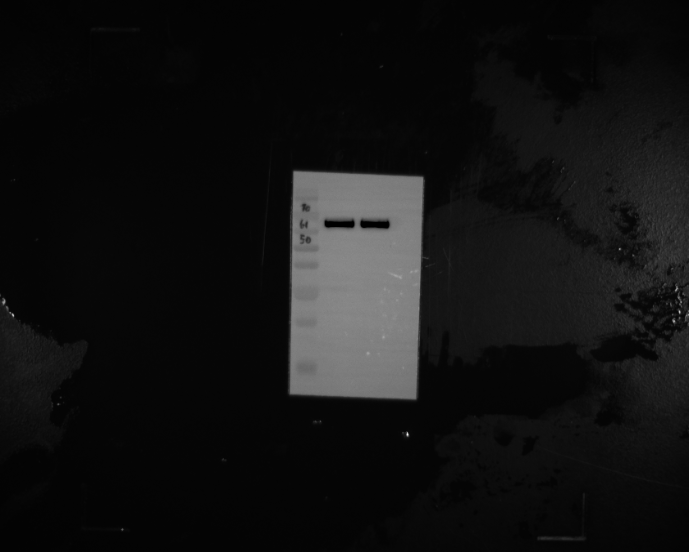


Figure 4G-CDKN1A


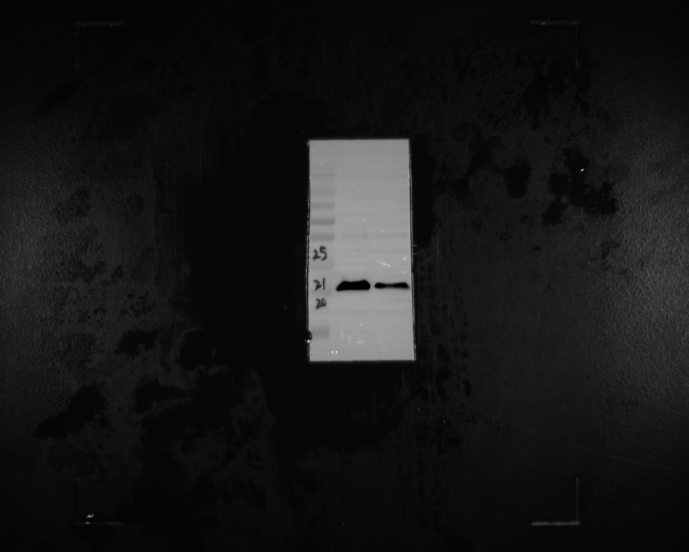


Figure 4G-IB-CDKN1A


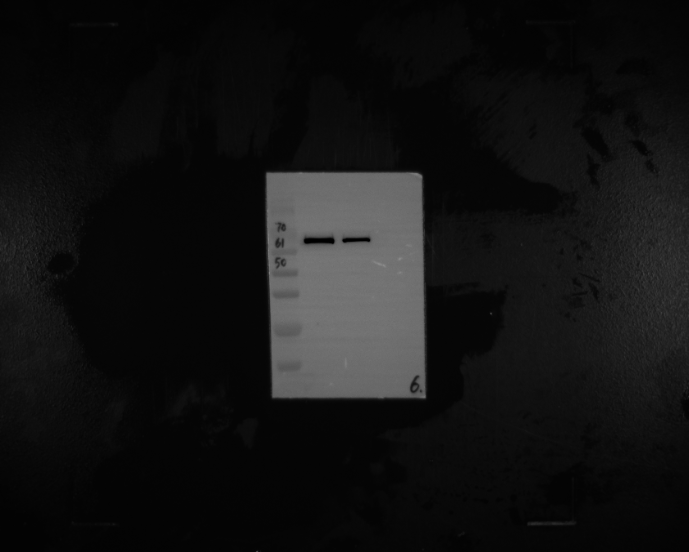


Figure 4G-SOCS5


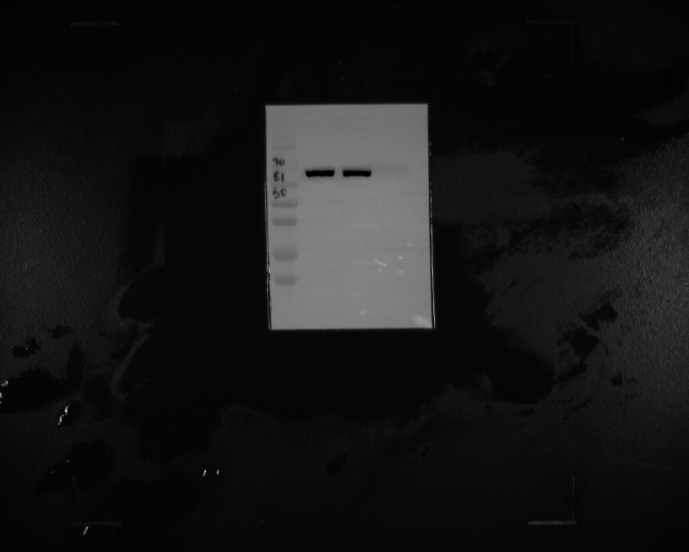


Figure 4H-CDKN1A


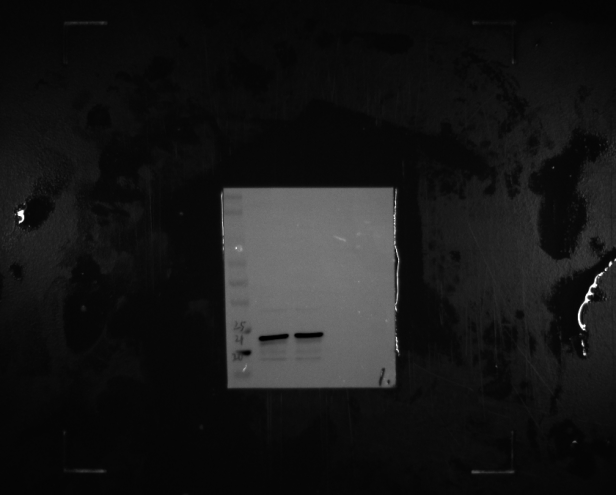


Figure 4H-SOCS5


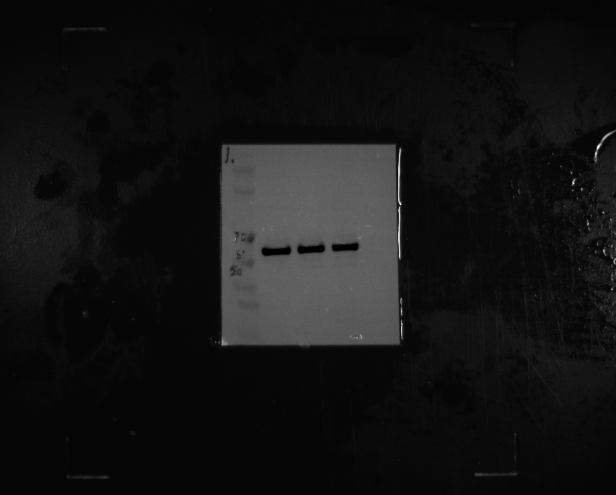


Figure 4I-NC-OE-CDKN1A


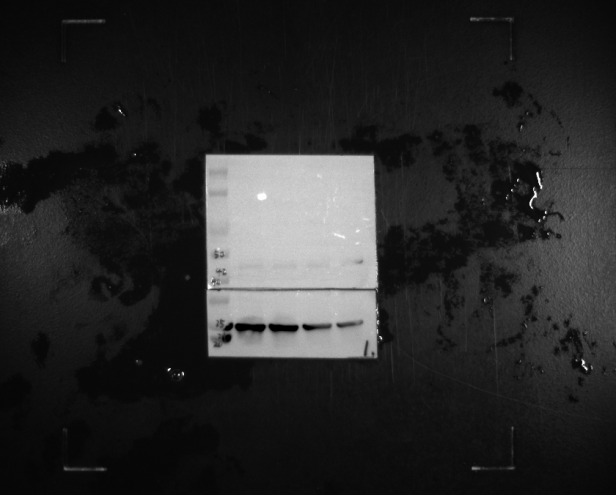


Figure 4I-NC-OE-β-actin


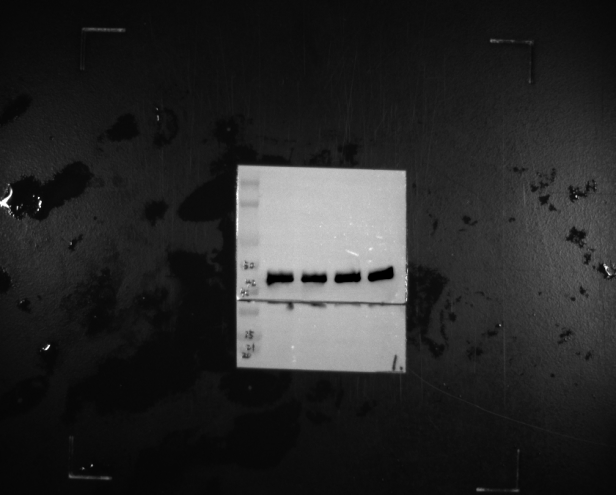


Figure 4I-SOCS5-OE-CDKN1A


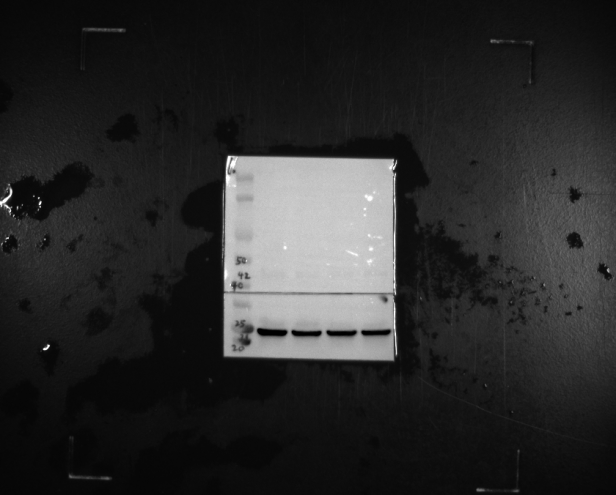


Figure 4I-SOCS5-OE-β-actin


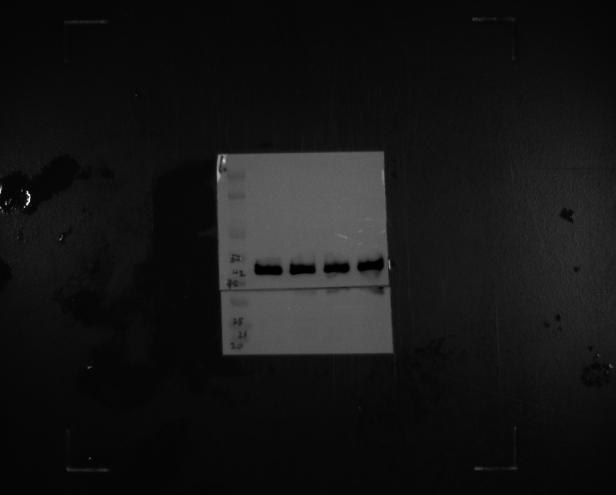


Figure 4J-CDKN1A


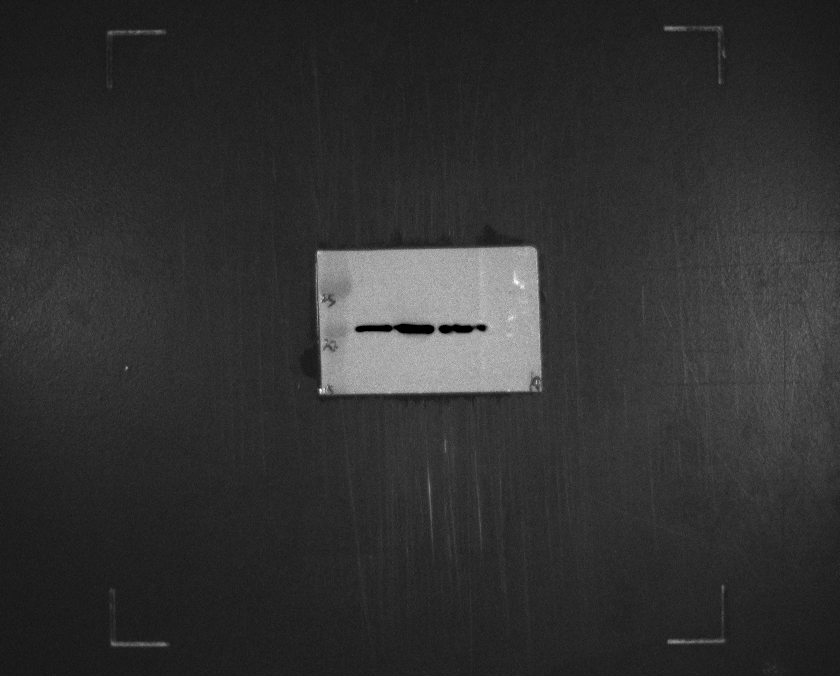


Figure 4J-SOCS5


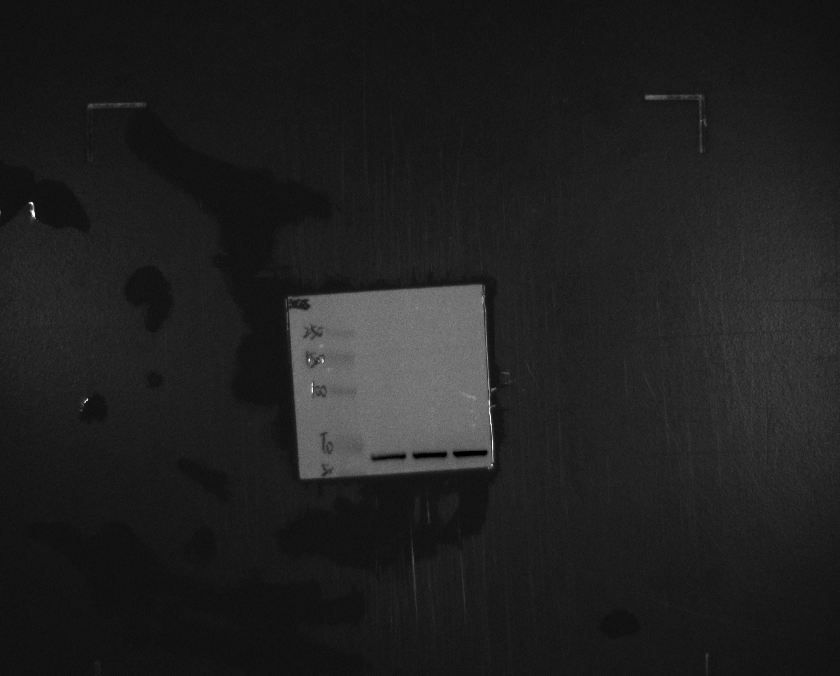


Figure 4J-β-actin


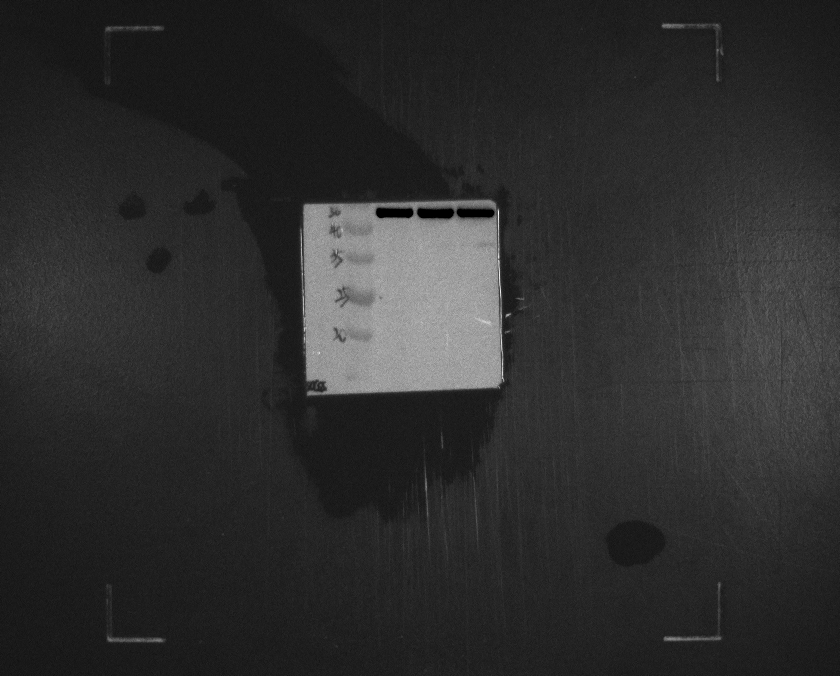


Figure 5B-POU2F1


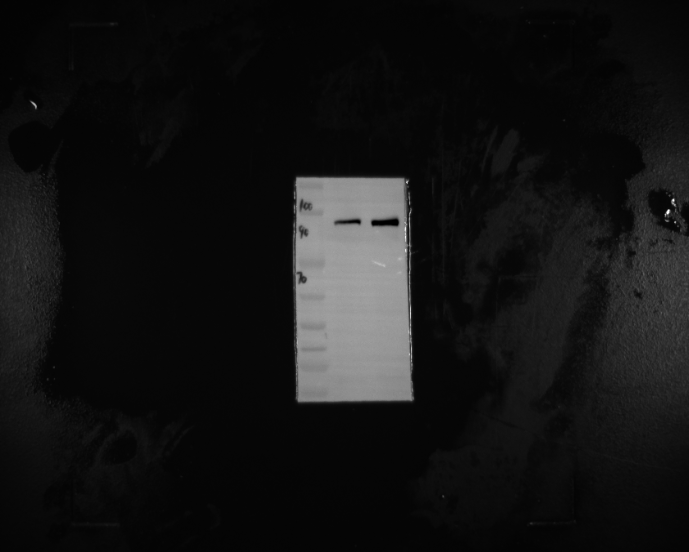


Figure 5B-β-actin


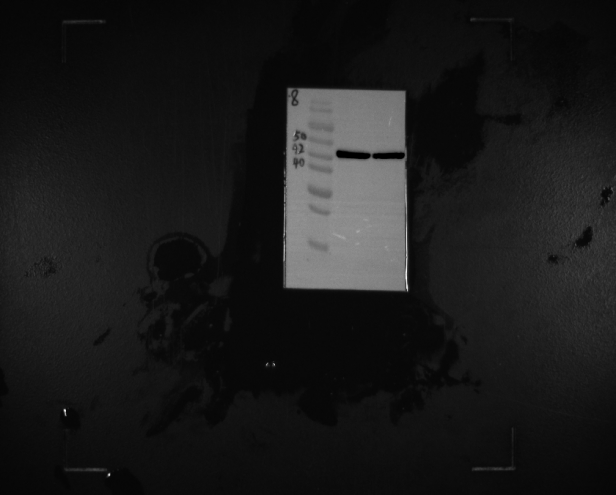


Figure 5E-POU2F1


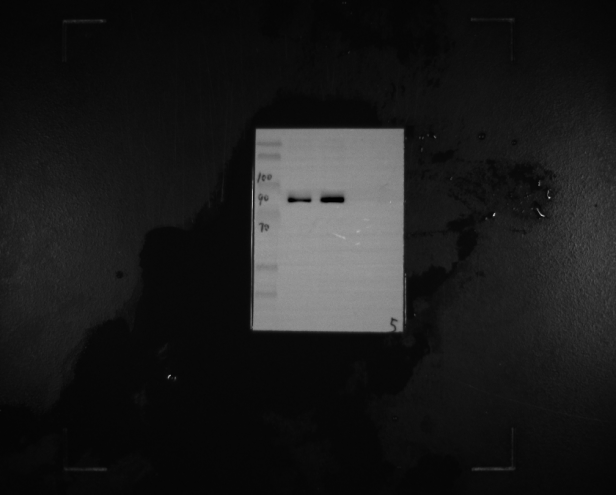


Figure 5E-β-actin


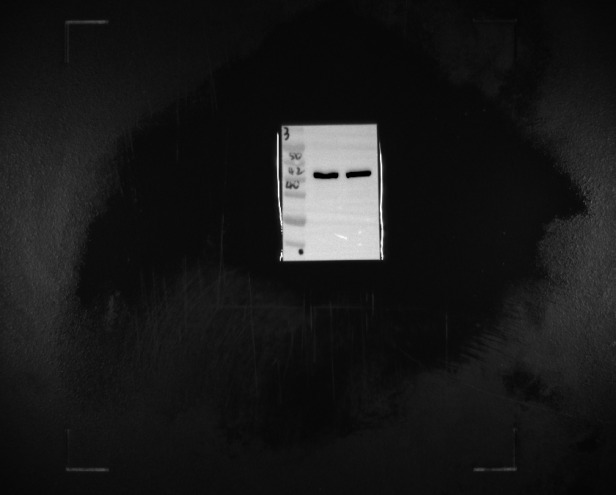


Figure 5H-CDKN1A


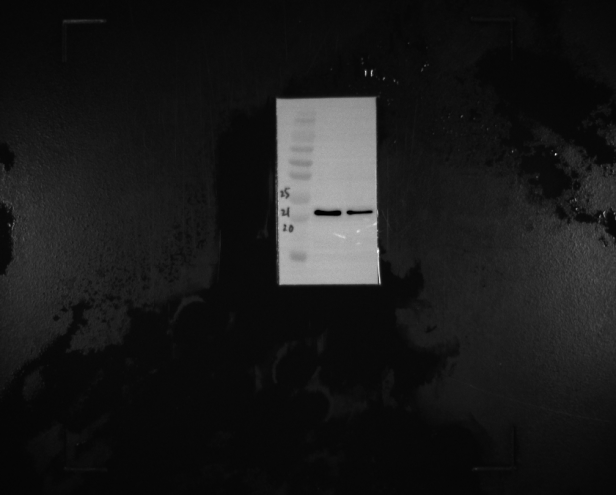


Figure 5H-POU2F1


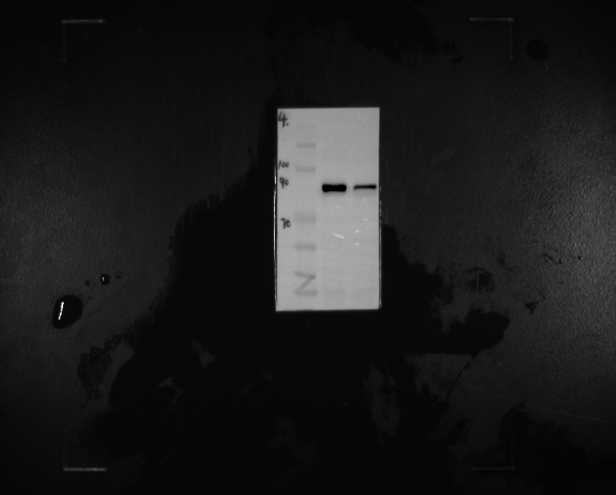


Figure 5H-SOCS5


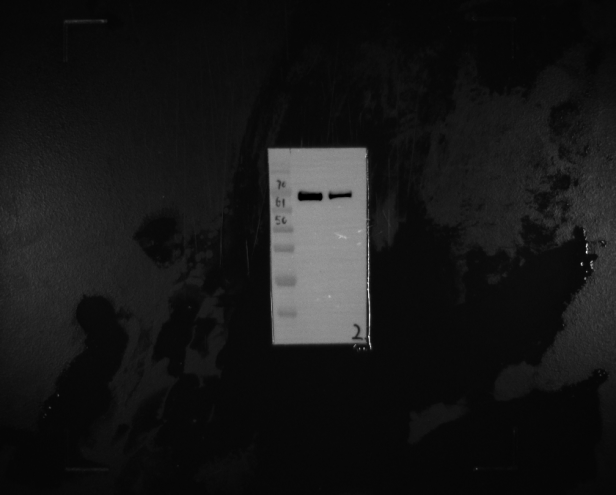


Figure 5H-β-actin


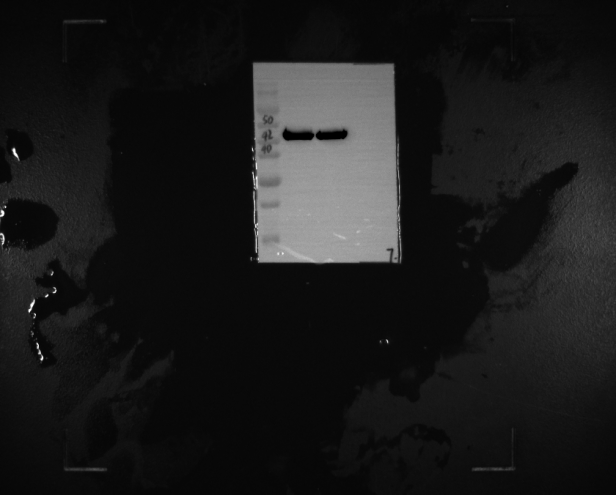


Figure 6B-CDKN1A


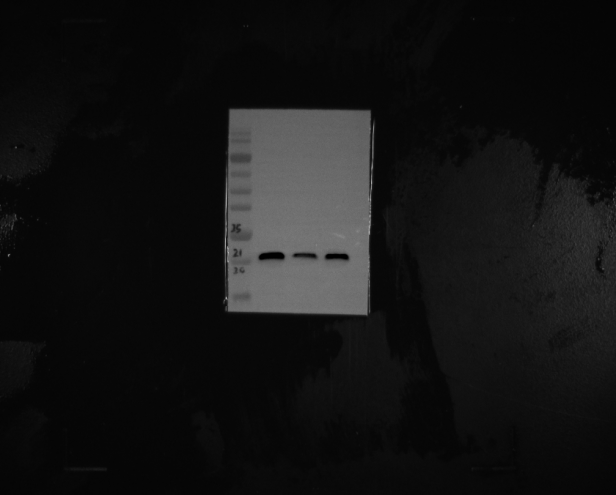


Figure 6B-POU2F1


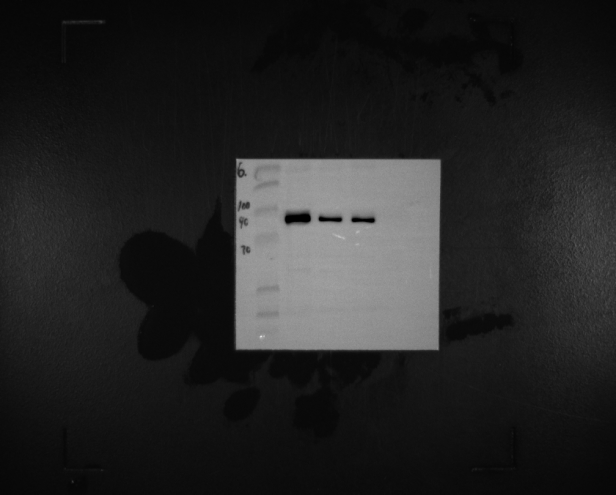


Figure 6B-SOCS5


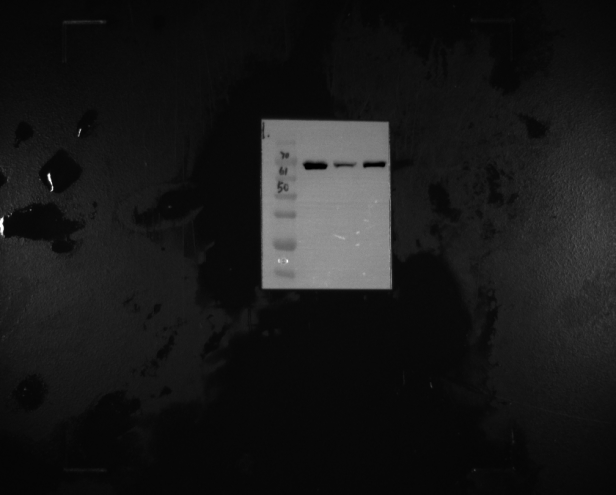


Figure 6B-β-actin


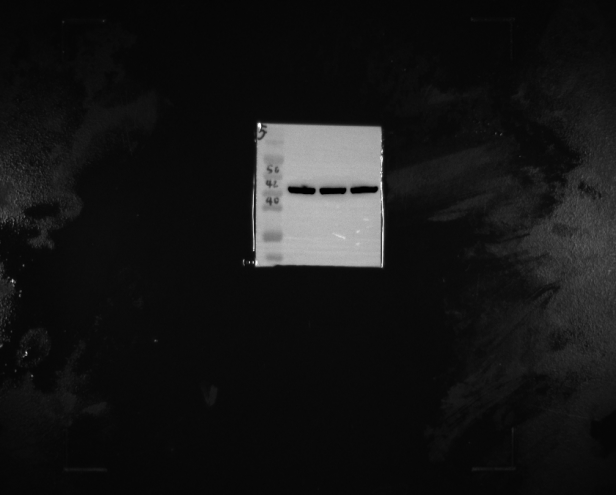


Figure 6G-P16


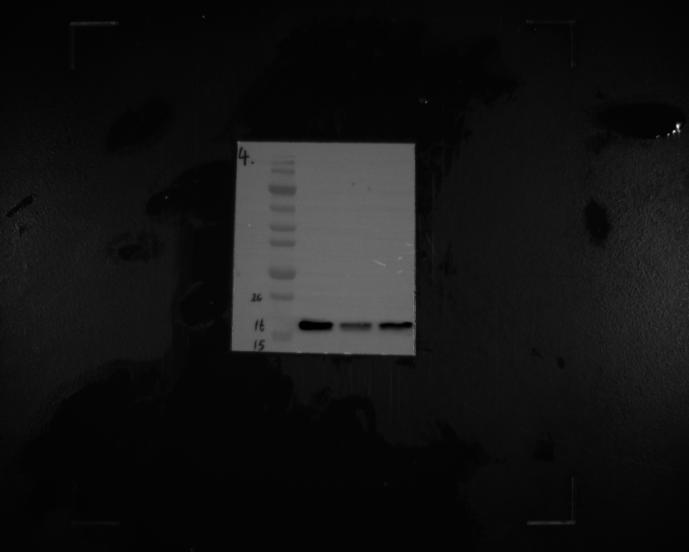


Figure 6G-P53


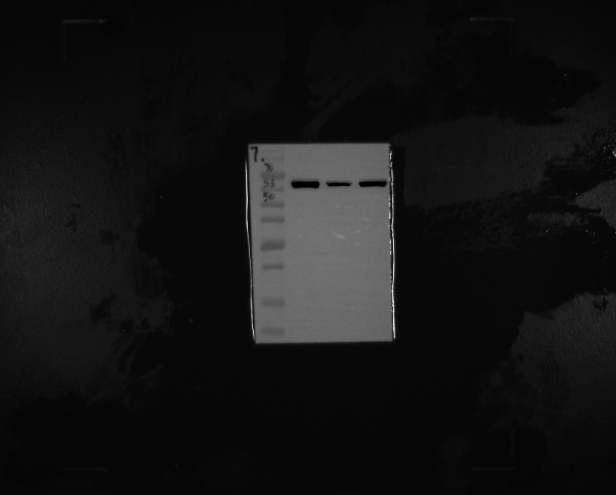


Figure 6G-β-actin


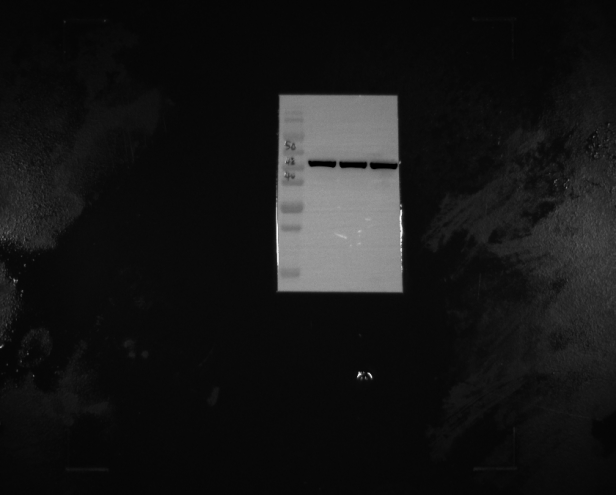


Figure 7B-CDKN1A


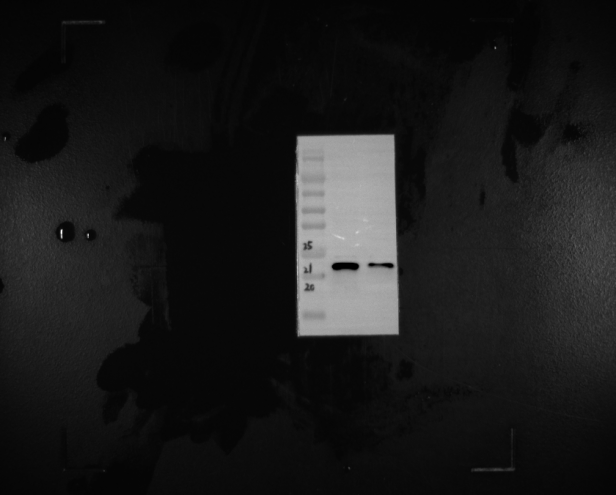


Figure 7B-POU2F1


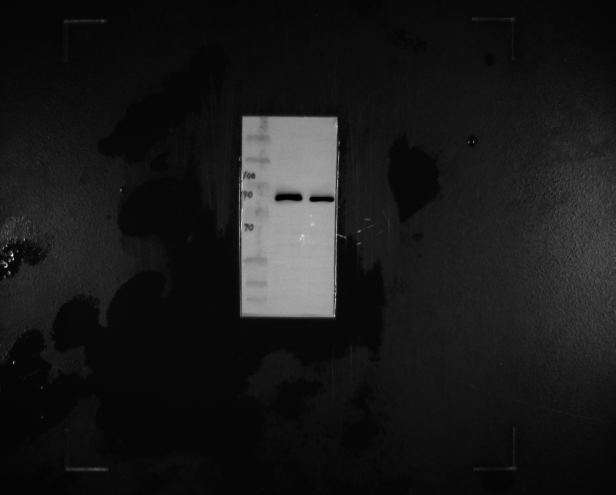


Figure 7B-SOCS5


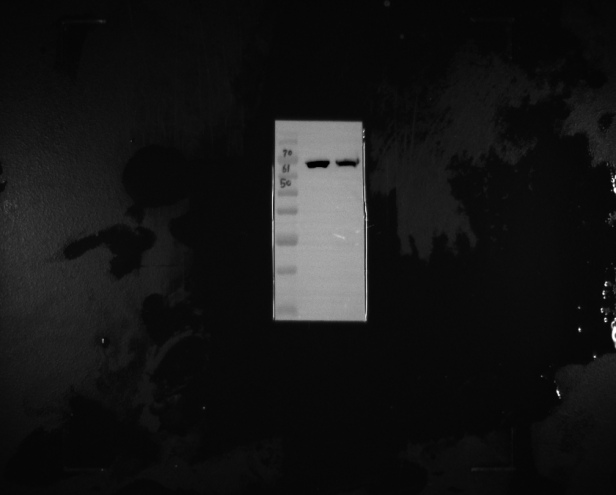


Figure 7B-β-actin


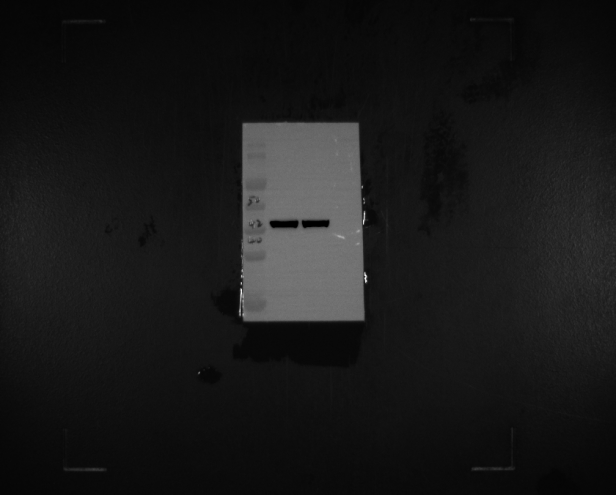


Figure 7J-P16


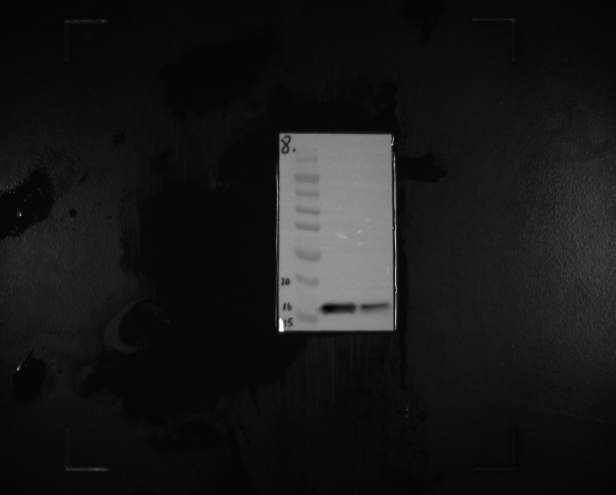


Figure 7J-P53


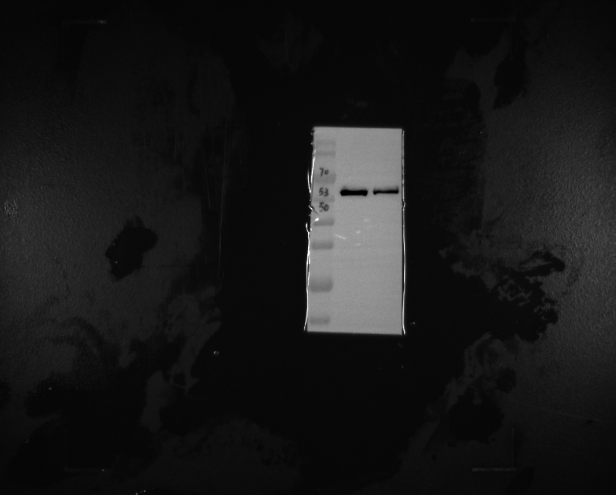


Figure 7J-β-actin


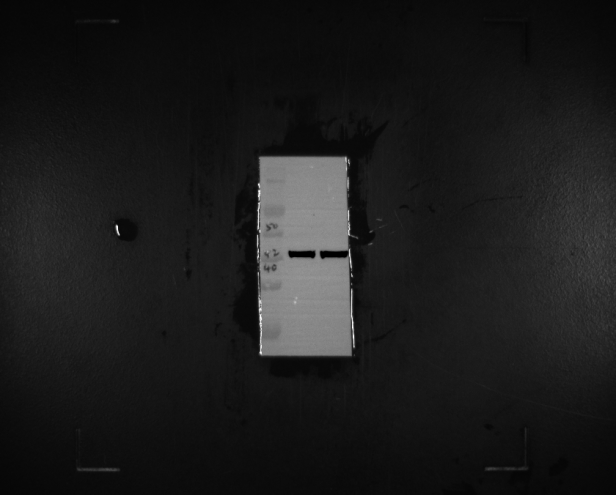


Figure S1B-SOCS5


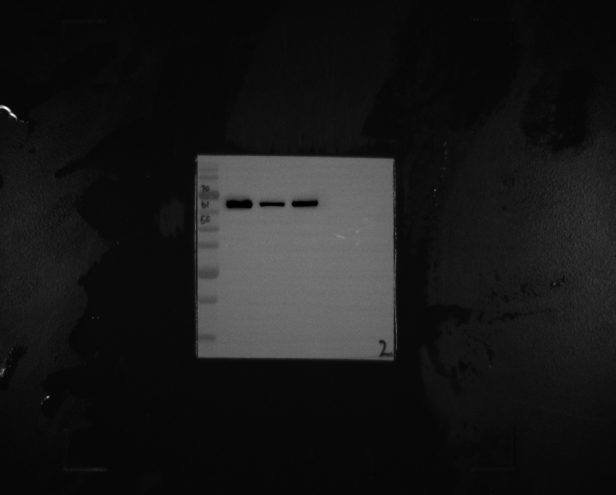


Figure S1B-β-actin


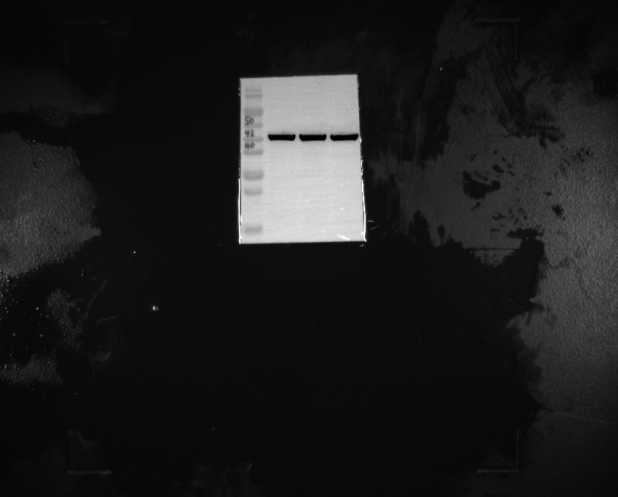


Figure S1D-SOCS5


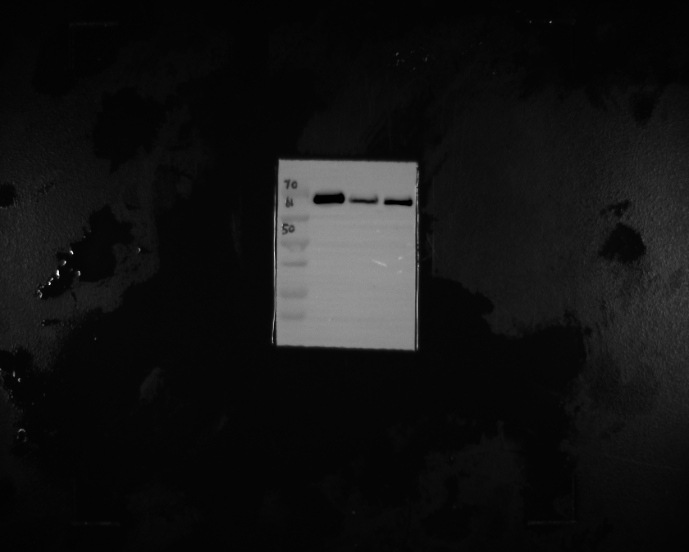


Figure S1D-β-actin


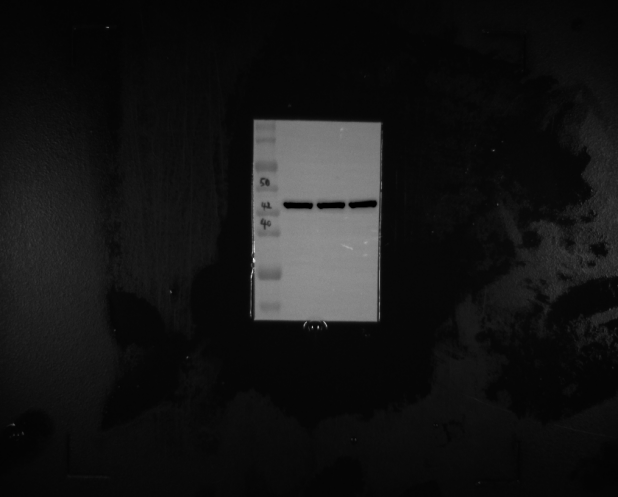


Figure S1F-CDKN1A


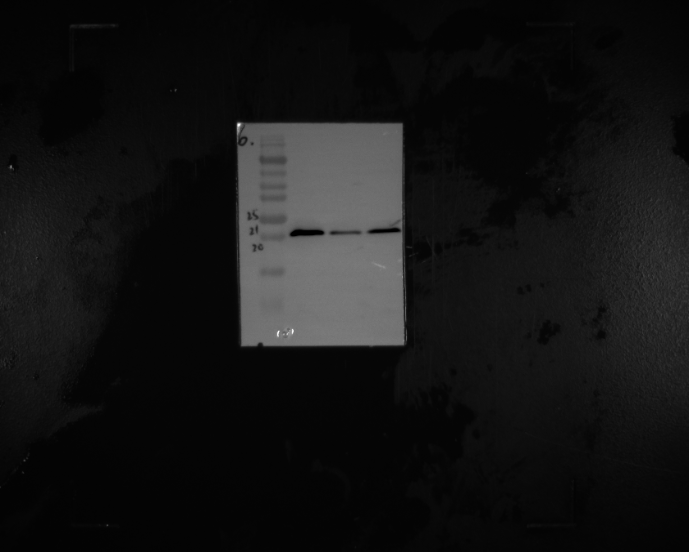


Figure S1F-β-actin


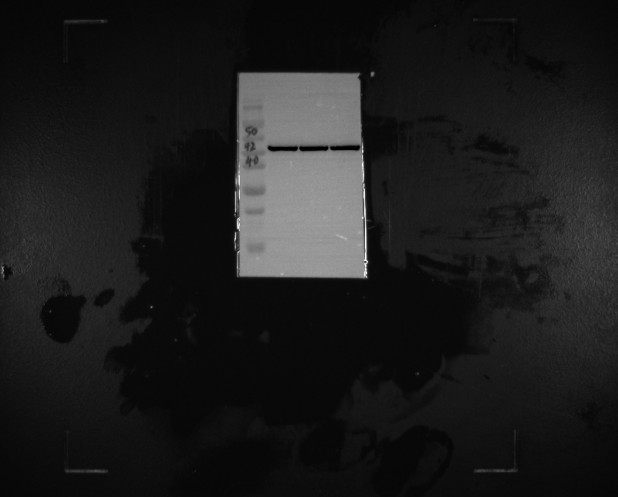


Figure S1H-POU2F1


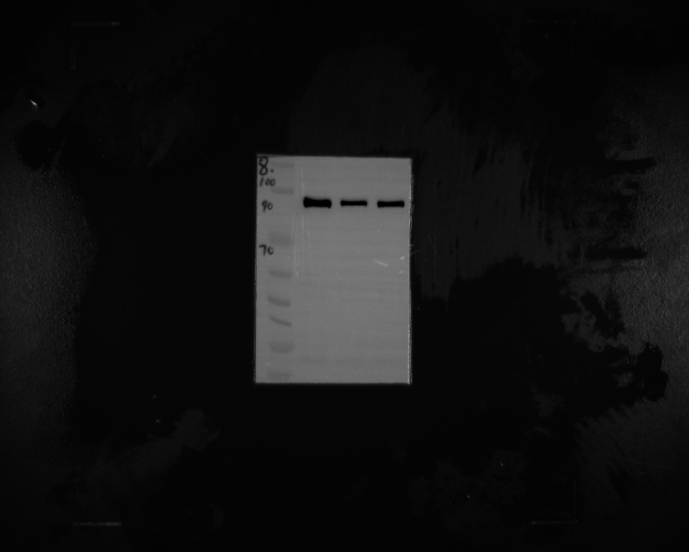


Figure S1H-β-actin


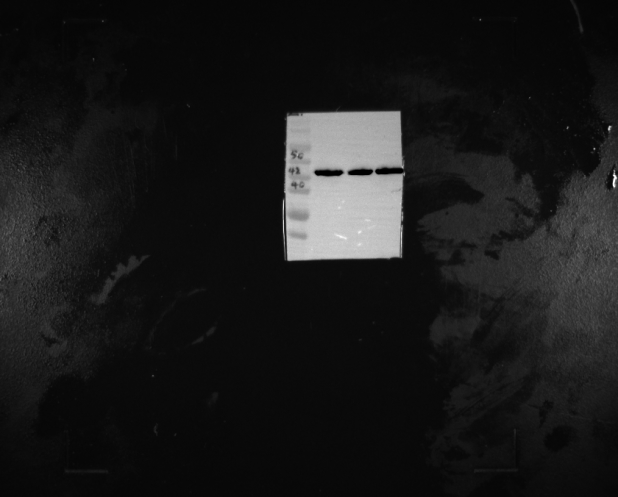


Figure S1J-POU2F1


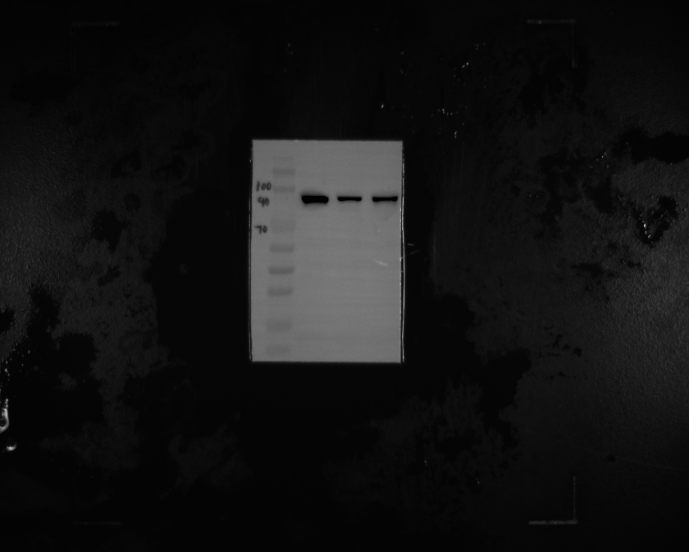


Figure S1J-β-actin


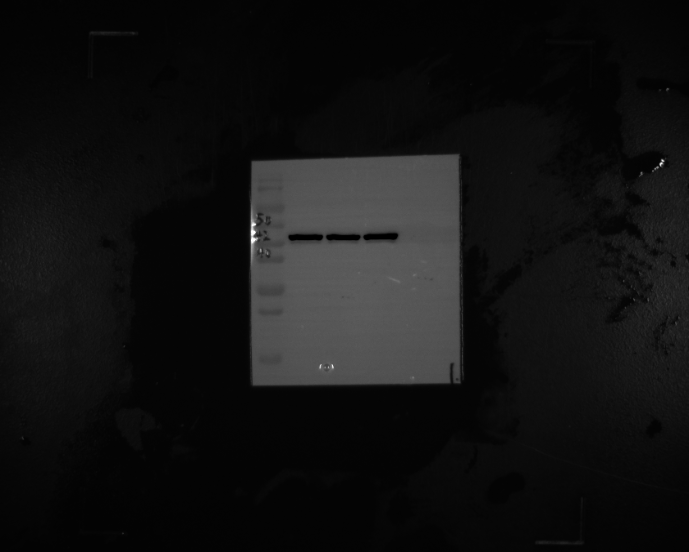


Figure S2B-SOCS5


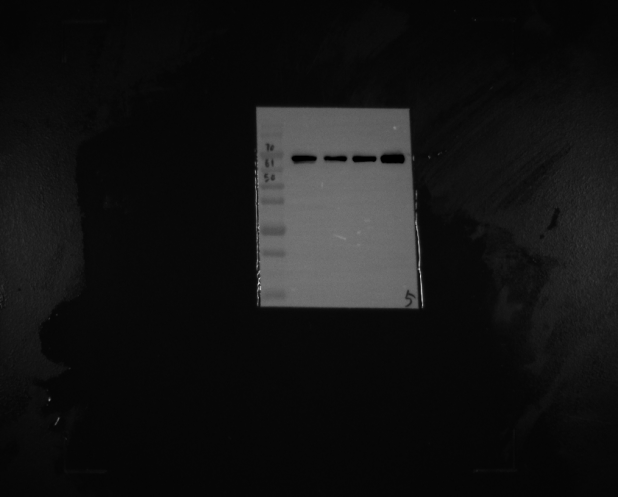


Figure S2B-β-actin


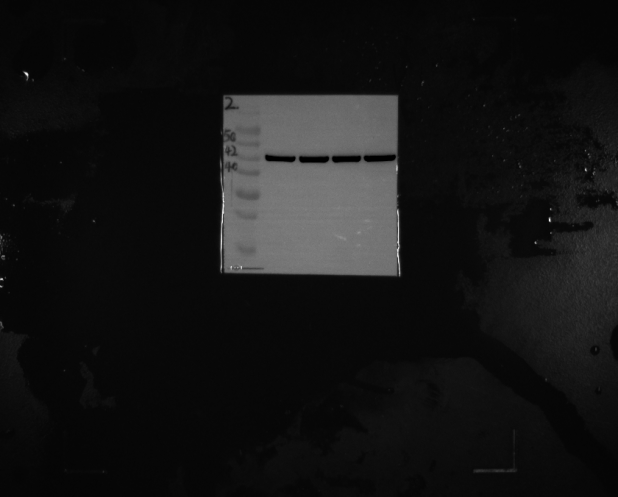


Figure S2F-SOCS5


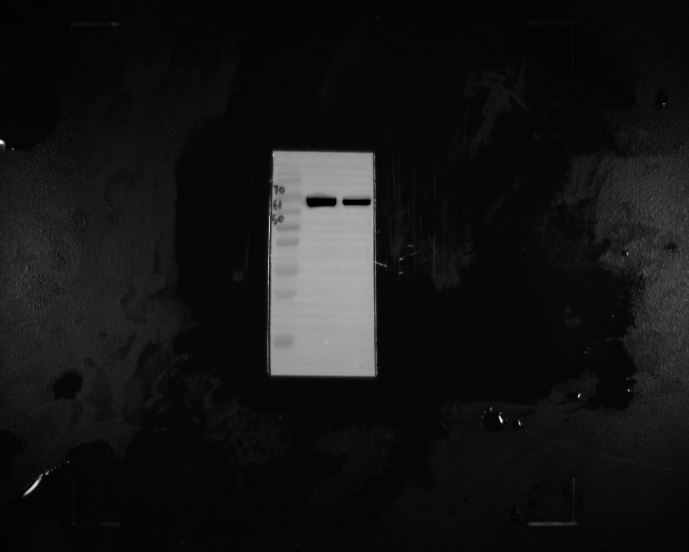


Figure S2F-β-actin


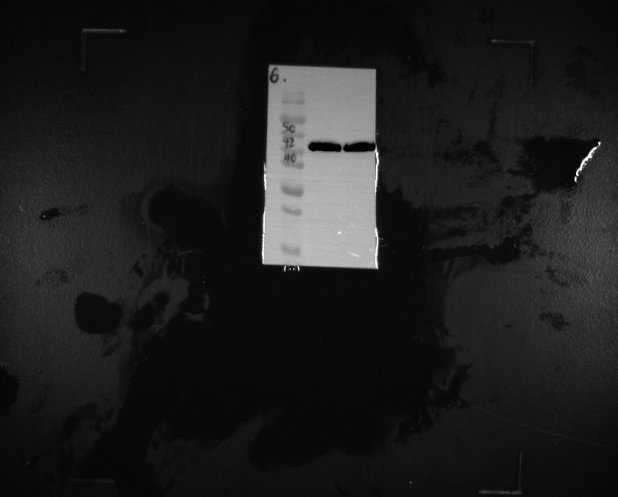


Figure S3H-CDKN1A


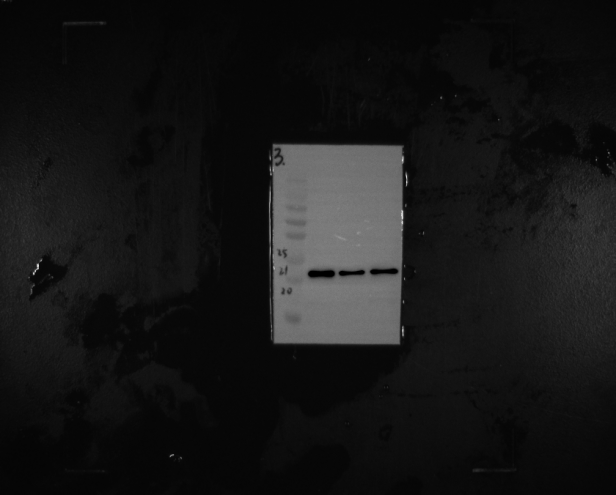


Figure S3H-SOCS5


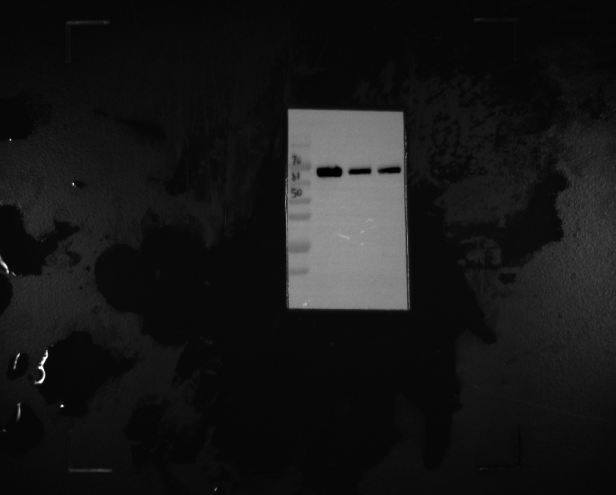


Figure S3H-β-actin


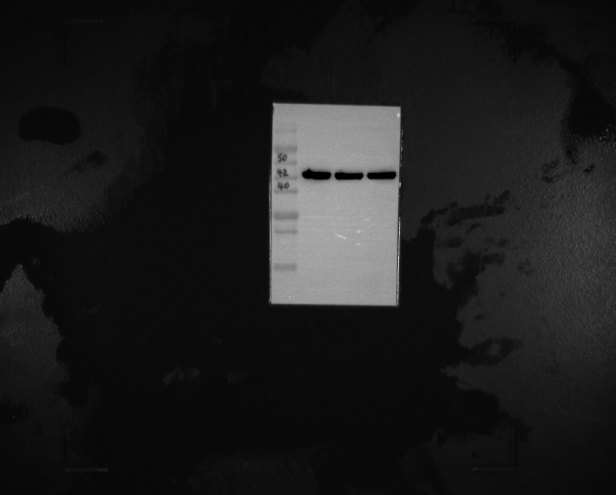


Figure S5-CDKN1A


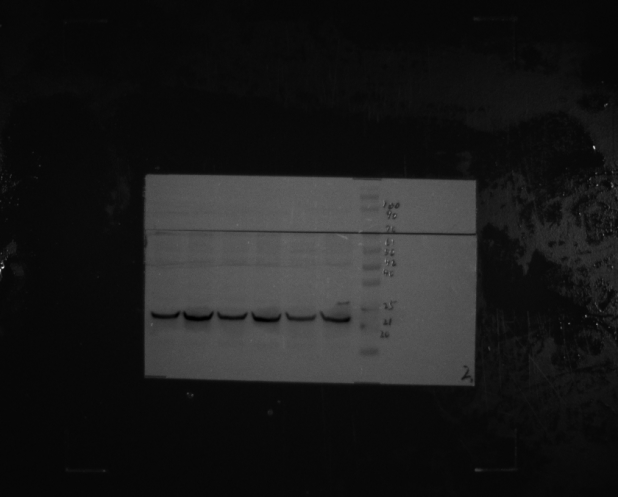


Figure S5-POU2F1


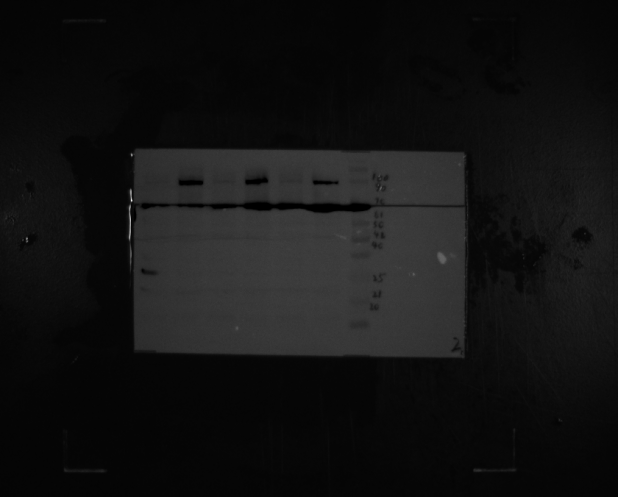


Figure S5-SOCS5

**
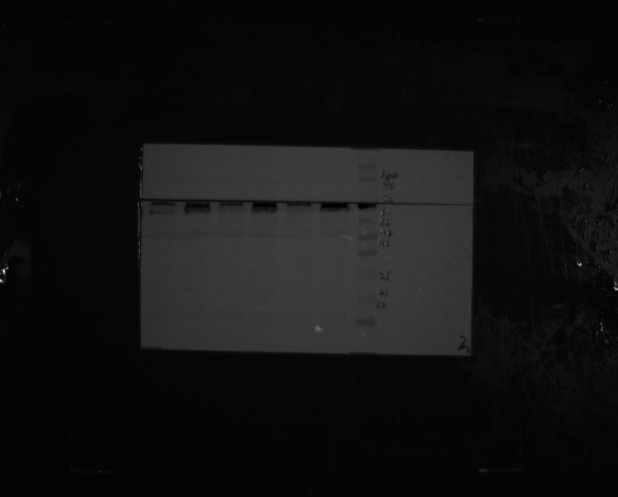
**

Figure S5-β-actin


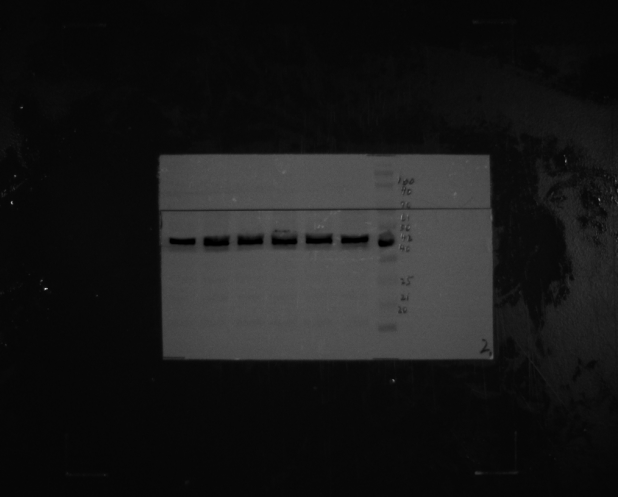

Supplement: Supplementary file 1 — Original Western blots [file 41420_2026_3011_MOESM1_ESM.docx]
