# Supplementary material for: Mechanistic insights into SOCS5-related DNA damage and cellular senescence in diabetic retinopathy
Source: Cell Death Discov. 2026 Apr 1;12:212. doi: 10.1038/s41420-026-03011-3 (PMC13168495; doi:10.1038/s41420-026-03011-3)
Supplement: Supplementary file 2 — Supplementary figures [file 41420_2026_3011_MOESM2_ESM.docx]

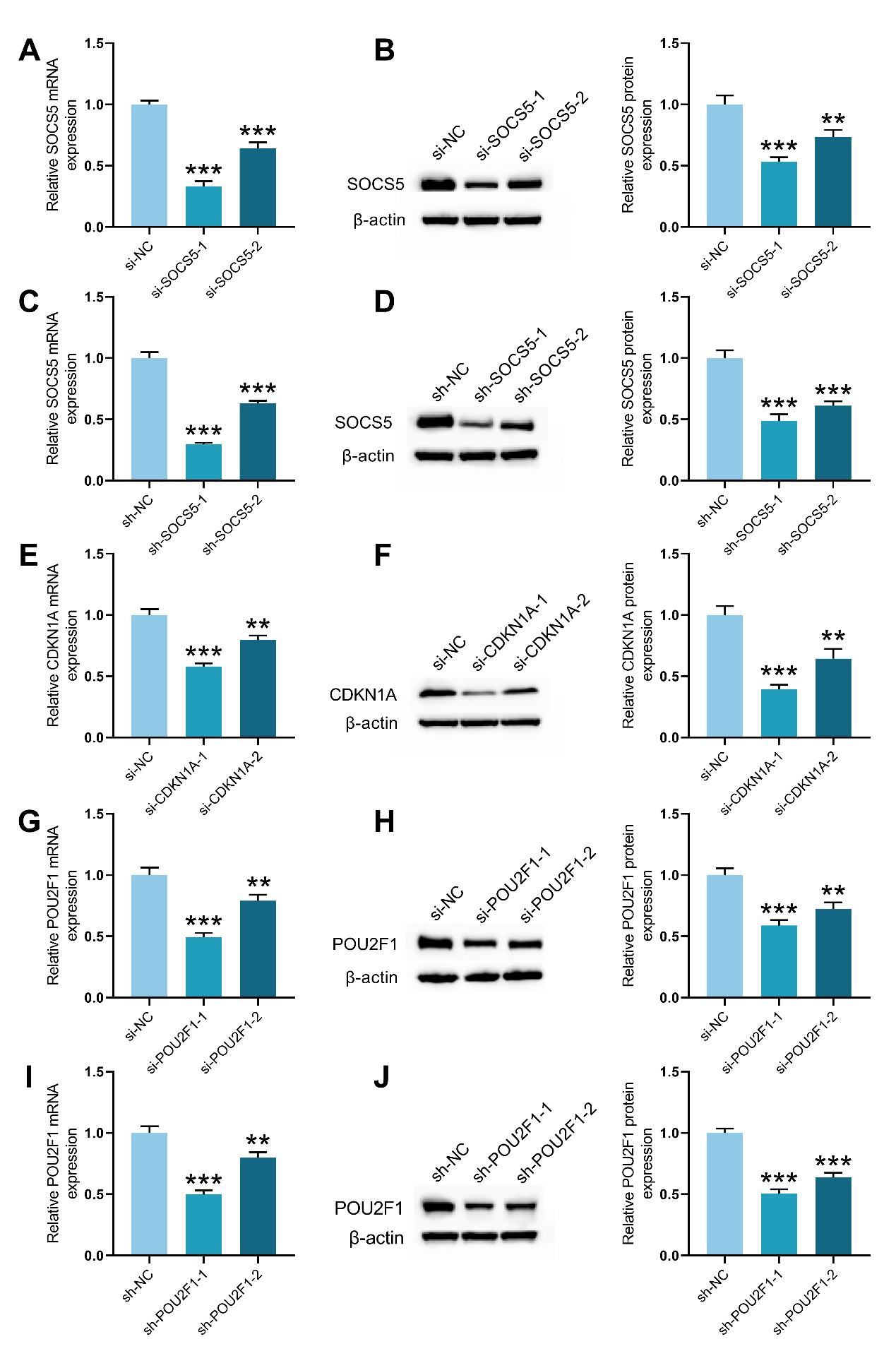
**Supplementary Figure 1 Validation of SOCS5, CDKN1A, POU2F1 interference efficiency**. (A-D) SOCS5 mRNA and protein levels was detected by qRT-PCR and WB in HRMECs transfected with si-SOCS5 and in retinal tissues of mice injected with AAV-sh-SOCS5. (E-F) CDKN1A mRNA and protein levels in HRMECs transfected with si-CDKN1A were measured by qRT-PCR and WB. (G-J) POU2F1 mRNA and protein levels were detected by qRT-PCR and WB in HRMECs transfected with si-POU2F1 and in retinal tissues of mice injected with AAV-sh-POU2F1. Student’s t tests were used for comparing two variables. One-way ANOVA test was used for multiple variable comparison. Data are from n independent biological replicates (n=3 independent cultures). **p* ˂ 0.05, ***p* ˂ 0.01, ****p* ˂ 0.001 vs si-NC/ sh-NC.


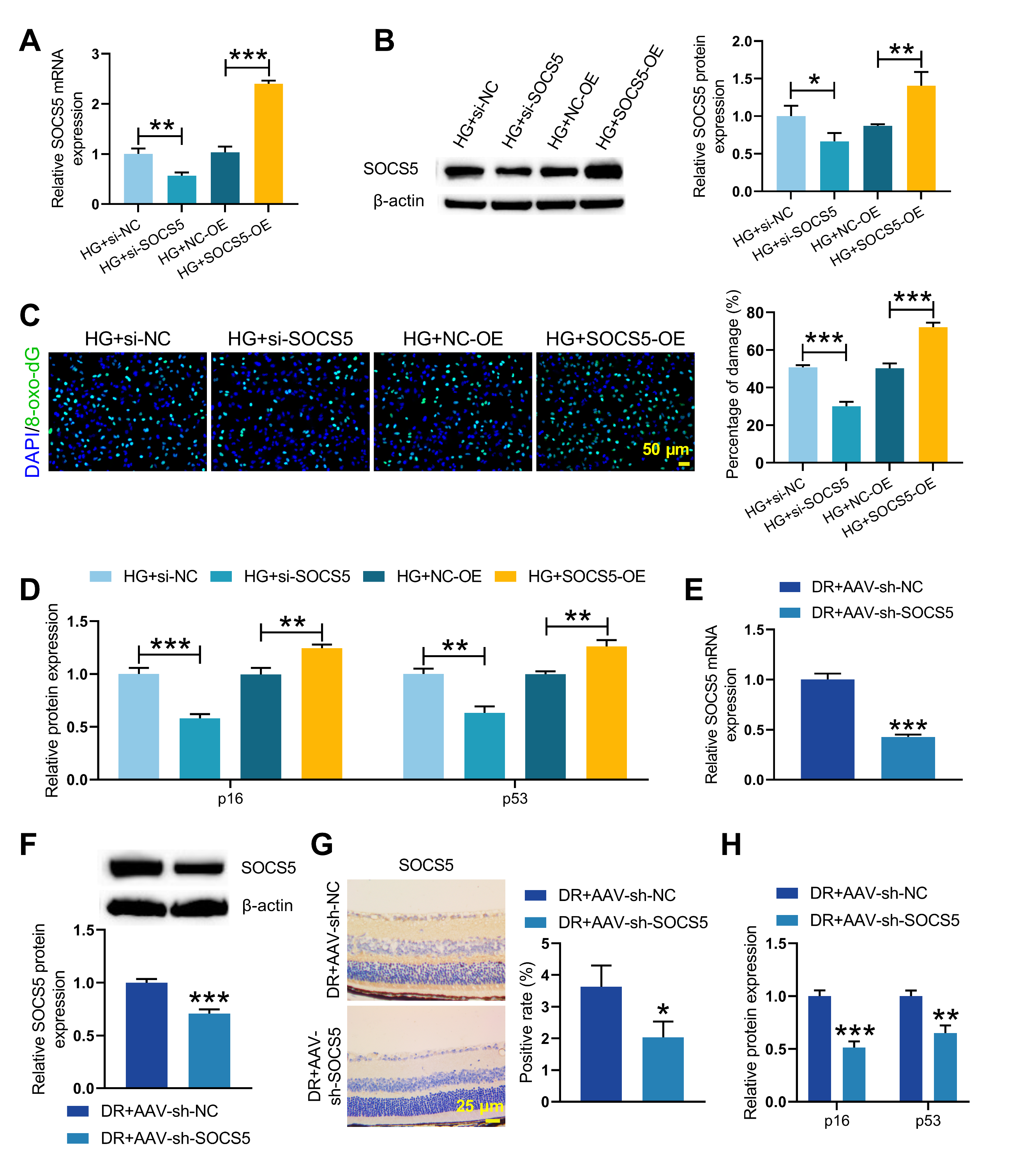


**Supplementary Figure 2 SOCS5 regulated DNA damage and senescence in DR models**. HRMECs were transfected with si-NC, si-SOCS5, NC-OE, or SOCS5-OE and then stimulated with HG for 48 h. (A-B) SOCS5 mRNA and protein levels in HRMECs were measured by qRT-PCR and WB. (C) Immunofluorescence staining of 8-oxo-dG assessing oxidative DNA damage in HRMECs. (D) WB analysis was performed to detect p53 and p16 protein levels in HRMECs. STZ-induced DR mice were injected with AAV-sh-NC or AAV-sh-SOCS5 (n=9 mice per group). (E-G) qRT-PCR, WB, and immunohistochemistry were performed to evaluate SOCS5 expression in retinal tissues of DR mice. (H) The impacts of sh-SOCS5 on senescence-related molecules p53 and p16 in retinal tissues from DR mice were verified by WB. Student’s t tests were used for comparing two variables. One-way ANOVA test was used for multiple variable comparison. Data are from n independent biological replicates (A-D, n=3 independent cultures; E-H, n=3 mice). **p* ˂ 0.05, ***p* ˂ 0.01, ****p* ˂ 0.001 vs HG+si-NC/ DR+AAV-sh-NC.


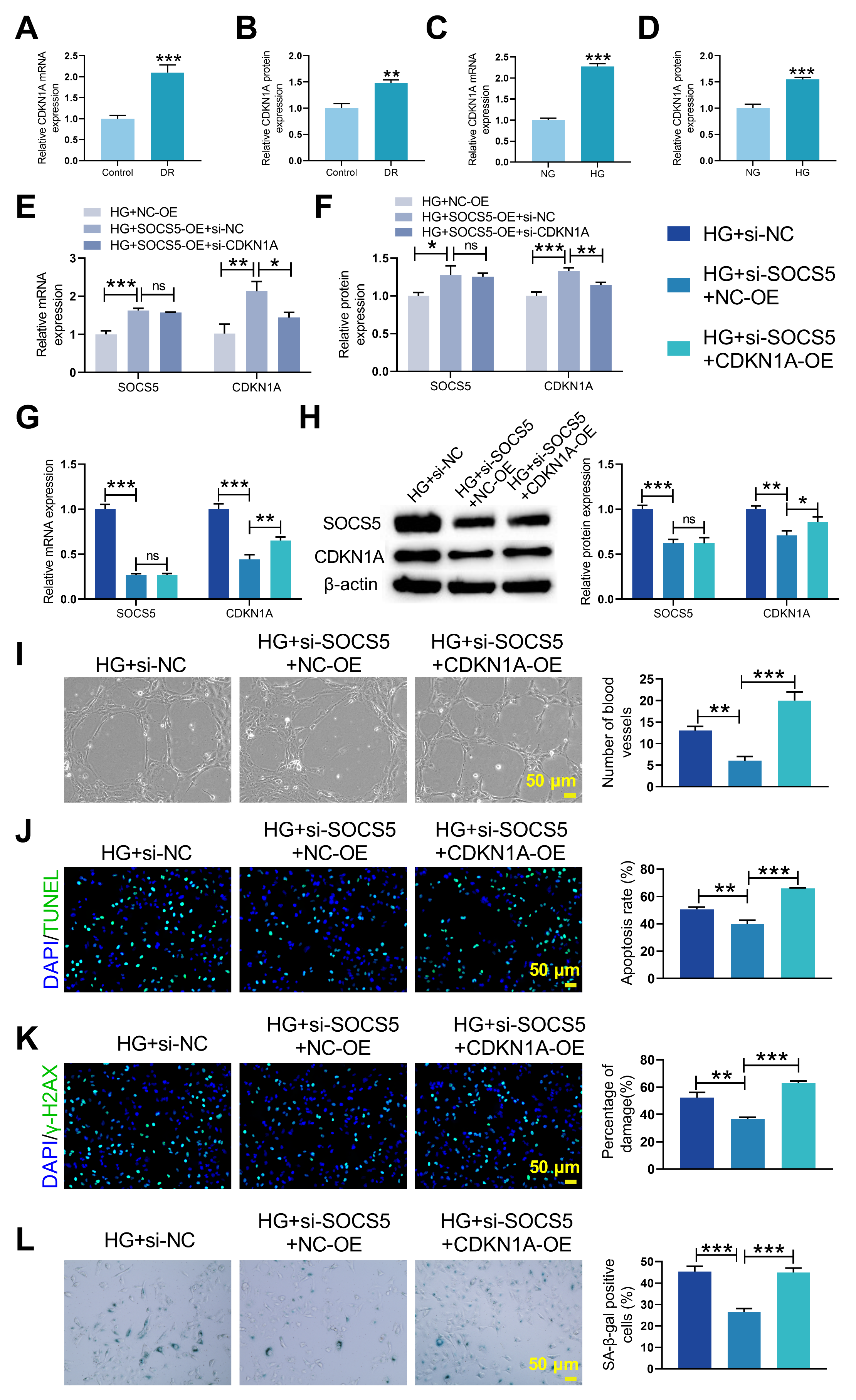


**Supplementary Figure 3 Detection of CDKN1A expression in mouse retinal tissues and HRMECs.** (A-B) CDKN1A mRNA and protein levels in retinal tissues from control and DR mice were measured by qRT-PCR and WB. (C-D) qRT-PCR and WB detection of CDKN1A expression in HRMECs treated with NG or HG. HRMECs were co-transfected with SOCS5-OE and/or si-CDKN1A followed by HG exposure. (E-F) qRT-PCR and WB were employed to measure SOCS5 and CDKN1A expression in HRMECs undergoing genetic manipulation. HRMECs were co-transfected with si-SOCS5 and/or CDKN1A-OE followed by HG exposure. (G-H) qRT-PCR and WB analysis of SOCS5 and CDKN1A expression in HRMECs undergoing genetic manipulation. (I) Matrigel tubule formation assay showing vascular formation in HRMECs after genetic manipulation. (J) TUNEL detection assessing apoptosis in HRMECs undergoing genetic manipulation. (K) γ-H2AX immunofluorescence assay was used to evaluate DNA damage in HRMECs after genetic manipulation. (L) SA-β-gal staining demonstrating senescence in HRMECs undergoing genetic manipulation. Student’s t tests were used for comparing two variables. Data are from n independent biological replicates (A-B, n=3 mice; C-L, n=3 independent cultures). **p* ˂ 0.05, ***p* ˂ 0.01, ****p* ˂ 0.001 vs Control/ NG/HG+si-NC/ HG+si-SOCS5+NC-OE.


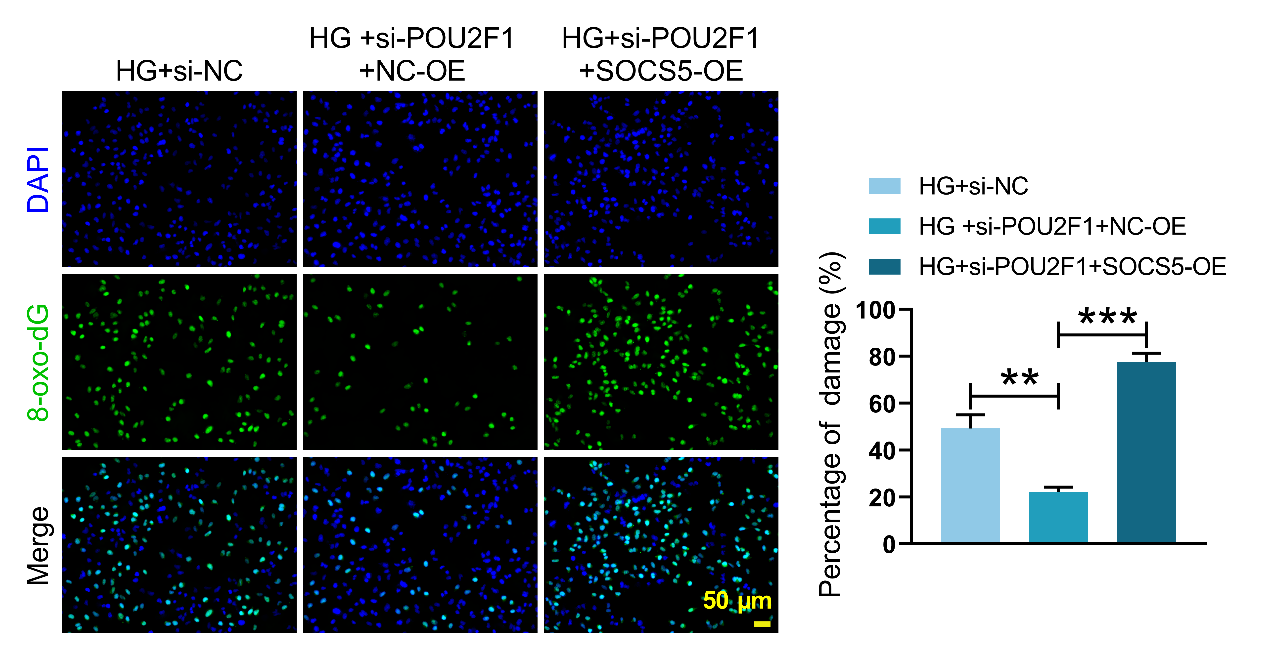


**Supplementary Figure 4 POU2F1 regulated oxidative DNA damage in HG-induced HRMECs through SOCS5.** HRMECs were co-transfected with si-POU2F1 and/or SOCS5-OE plasmids followed by HG stimulation for 48 h. The oxidative DNA damage in HRMECs was detected by 8-oxo-dG immunofluorescence staining. One-way ANOVA test was used for multiple variable comparison. Data are from n independent biological replicates (n=3 independent cultures). **p* ˂ 0.05, ***p* ˂ 0.01, ****p* ˂ 0.001 vs HG+si-NC/ HG+si-POU2F1+NC-OE.

**
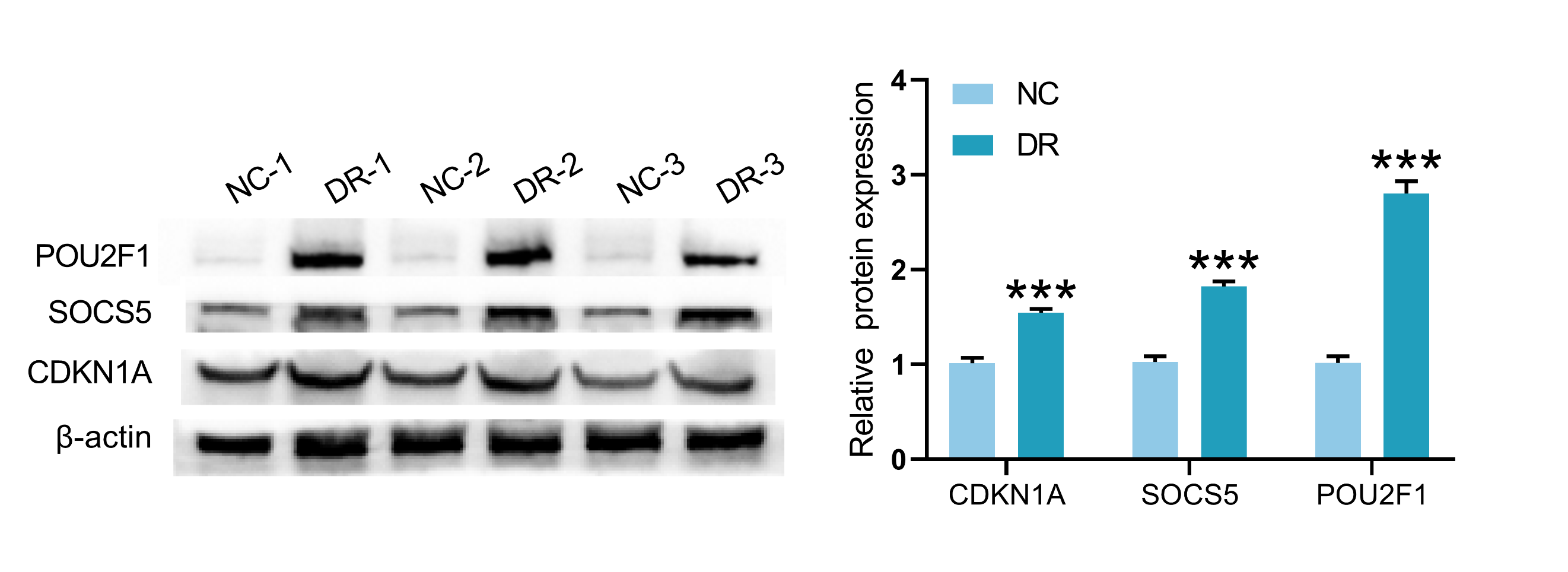
**

**Supplementary Figure 5 The expression levels of CDKN1A, SOCS5, and POU2F1 were elevated in the peripheral blood of patients with DR.** WB analysis was performed to detect the protein expression of CDKN1A, SOCS5, and POU2F1 in the peripheral blood of normal control (NC) subjects and patients with DR. Student’s t tests were used for comparing two variables. Data are from n independent biological replicates (n=3 independent clinical samples). **p* ˂ 0.05, ***p* ˂ 0.01, ****p* ˂ 0.001 vs NC.
