# Supplementary figures and images for: Mechanistic insights into SOCS5-related DNA damage and cellular senescence in diabetic retinopathy
Source: Cell Death Discov. 2026 Apr 1;12:212. doi: 10.1038/s41420-026-03011-3 (PMC13168495; doi:10.1038/s41420-026-03011-3)

| Sample File | Sample Name | Panel           | OS          | SQ          |
|-------------|-------------|-----------------|-------------|-------------|
| Sample18AFA | HRCEC       | STR Profile 1-F | <div></div> | <div></div> |

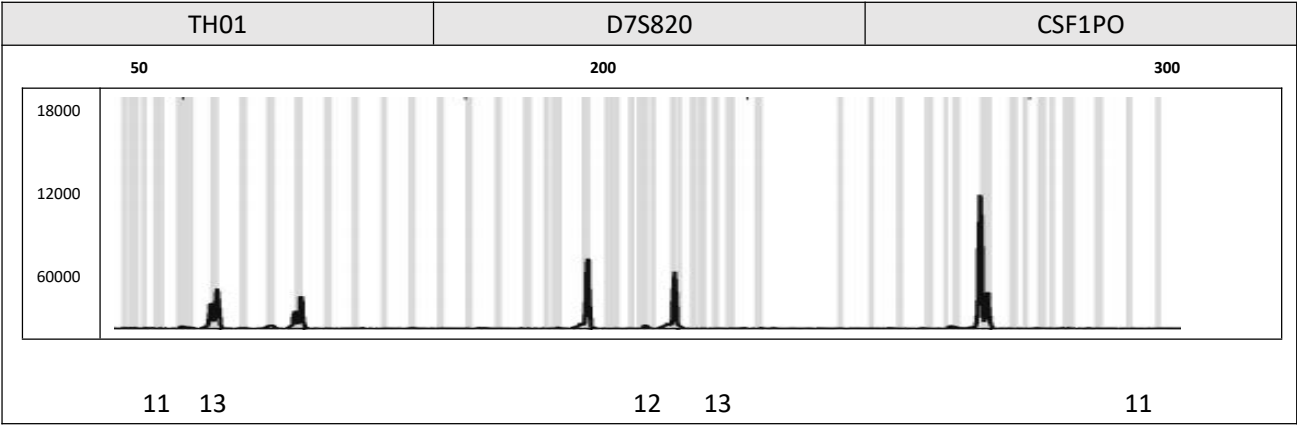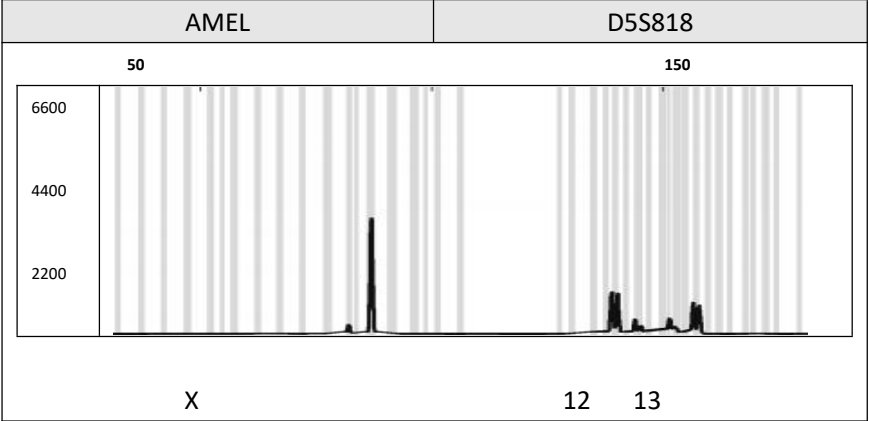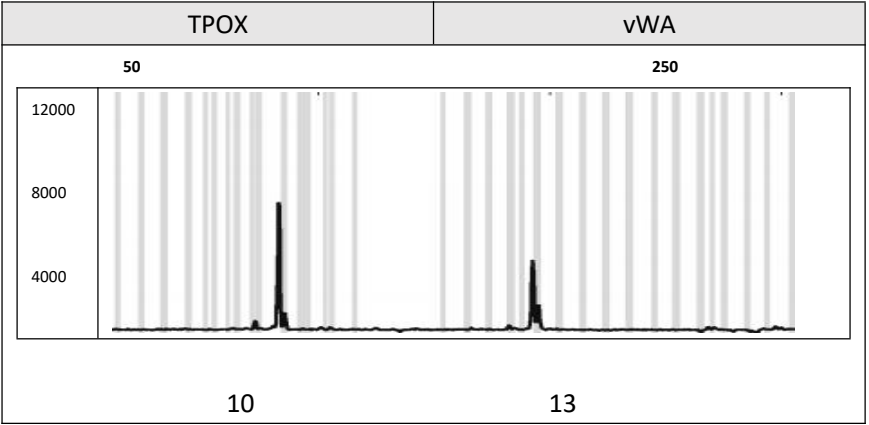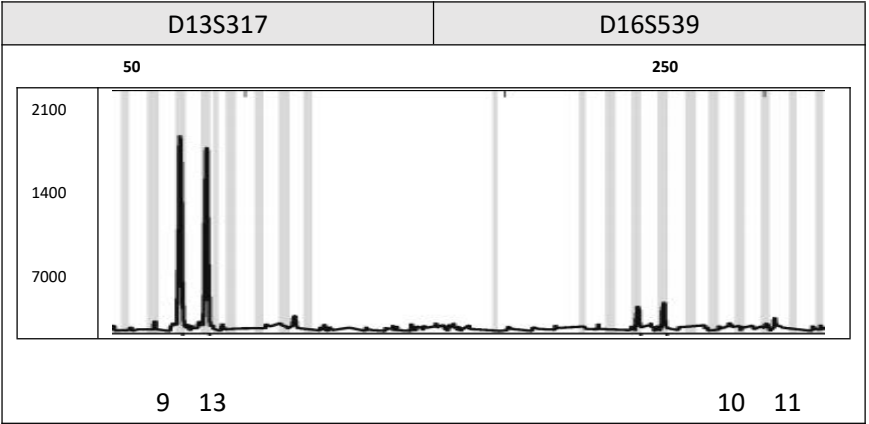

Supplement: Supplementary file 3 — File 1-STR-HRCEC [file 41420_2026_3011_MOESM3_ESM.pdf]
